# Visiting a loved one in the ICU with the aid of dedicated booklets is associated with reduced separation anxiety in children and adolescents

**DOI:** 10.1186/s13034-025-00906-4

**Published:** 2025-06-06

**Authors:** Giulia Lamiani, Federica Bonazza, Michela Maxia, Massimo Walter Rivolta, Giovanni Mistraletti, Elena Vegni

**Affiliations:** 1 Dipartimento di Scienze della Salute, Università degli Studi di Milano, Milan, Italy; 2 UOC Psicologia Clinica, ASST Santi Paolo e Carlo, Milan, Italy; 3 Dipartimento di Informatica “Giovanni Degli Antoni”, Università degli Studi di Milano, Milan, Italy; 4 Ospedale Civile di Legnano, ASST Ovest Milanese, Legnano,, Italy; 5 Dipartimento di Fisiopatologia Medico-Chirurgica e dei Trapianti, Università degli Studi di Milano, Milan, Italy

**Keywords:** ICU visiting policies, Children, Psychological well-being, Family members, Family-centered care, Critical illness

## Abstract

**Background:**

Opening visits in Intensive Care Units (ICU) to children and adolescents encounters fears and resistance. The aim of this study was to evaluate the impact on children’s psychological well-being of a prepared visit to family members hospitalized in the ICU.

**Methods:**

A quasi-experimental, pre-post-intervention design was implemented in 3 ICUs where children were allowed to visit. Children (7–17 years) with a close family member (parent, sister/brother, grandparent) hospitalized in ICU on mechanical ventilation for ≥ 48 h were eligible to participate. Within three days of the family member’s admission, children completed online pre-questionnaires to assess separation anxiety, Post Traumatic Stress Disorder (PTSD) symptoms and positive and negative affects. After completion of pre-questionnaires, if parents and children expressed the desire to visit, an age-appropriate educational booklet, which was purposefully developed, was offered and the visit was planned and prepared by ICU staff. After the visit, children completed the same questionnaires. Children who did not visit completed the same questionnaires a week after having completed the pre-questionnaires.

**Results:**

25 children (8 boys; mean age = 11.3; SD = 2.4) completed pre and post-questionnaires. Children who visited (*n* = 17) were older than children who did not visit (U = 109.000; *p* = 0.016), presented higher separation anxiety (U = 102.500; *p* = 0.043) and lower positive affects (U = 30.000; *p* =0.027) at pre-questionnaires. Children who visited reported a decrease in separation anxiety (Median-pre = 8; Median-post = 5; *p* = 0.004), PTSD symptoms (Median-pre = 25; Median-post = 18; *p* = 0.008) and an increase in positive affect (Median-pre = 33; Median-post = 34; *p* = 0.004) at post-questionnaires. Children who did not visit reported a decrease in PTSD symptoms (Median-pre = 13; Median-post = 10; *p* =.018) at post-questionnaires. Compared to children who did not visit, children who visited reported a decrease in separation anxiety (*U* = 22.500; *p* =.006) at post questionnaires. All children who visited (100%) were satisfied and 94% perceived the educational booklets as useful.

**Conclusion:**

Findings suggest that a prepared visit to family members in ICU may decrease children’s separation anxiety, without evident harm.

**Supplementary Information:**

The online version contains supplementary material available at 10.1186/s13034-025-00906-4.

## Background

Admission to the Intensive Care Unit (ICU) can cause stress, anxiety, and depression not only in patients but also in their family members [1, 2]. Several studies highlighted the importance for family members to maintain physical closeness with the patients admitted to the ICU by visiting them without physical or time restrictions [3, 4].

Studies have shown that adopting an open ICU visiting policy does not expose patients to additional risk for infection [5]. On the contrary, it was proven to be useful in promoting patient’s well-being, leading to a reduction in cardiovascular complications, anxiety, delirium, hormonal stress markers, and an improvement in sleepn [6, 7]. Family members also experienced a decrease in stress and anxiety levels in ICUs with unrestricted visiting hours [8]. Based on this evidence, recommendations have been made to abolish restricted visiting hours and reduce unnecessary barriers between patients and family members [9, 10, 11]. However, the process of opening ICUs to family members often encounters resistance, especially when the visitors are children and adolescents. The main concerns of parents and clinicians are related to the potentially traumatic aspects of the visit and the fear of not knowing how to manage the emotional and organizational aspects of the visit [12].

Some qualitative studies have shown that, for children and adolescents, the experience of visiting their loved one is not only characterized by fear, but also by curiosity, relief, and reassurance [13, 14]. This evidence is consistent with Bowlby’s attachment theory (1969) [15] which emphasizes how maintaining physical proximity to attachment figures, in the presence of a physical or psychological threat, is an innate need for children because it helps to regulate emotions and enhance psychological security [16]. A recent review of existing literature [17] found that the experience of visiting enabled children and adolescents to gain a deeper understanding of reality and preserve their relationships with family members. Children/adolescent’s visits to the ICU seemed to have positive effects, provided there is preparation and facilitation [17]. Recently, guidelines for clinicians on how to prepare and support children and adolescents who wish to visit their family members in the ICU were published [18]. However, there is a lack of quantitative evidence on the psychological impact of minors visiting the ICU [19].

The aim of this study was to assess if a prepared visit to family members in the ICU could enhance children and adolescents’ psychological well-being in term of separation anxiety, post-traumatic stress, and positive and negative affects. Based on Bowlby’s attachment theory [15], we hypothesized a significant decrease in separation anxiety and negative emotions for children/adolescents who visited their family member, compared to those who did not visit. As the visit was prepared and facilitated, we hypothesized a significant decrease in post-traumatic stress symptoms for children/adolescents who visited compared to those who did not. Lastly, as the situation that children/adolescents were experiencing was delicate, we did not expect any change in positive emotions.

## Methods

This study is part of the MINVITI “Minors visiting Intensive Care Unit” project (original name: “MINori in VIsita in Terapia Intensiva”), which aims to humanize ICU care and promote family-centered care. As a part of this project, educational booklets were developed by an interdisciplinary group composed of psychologists, ICU physicians and nurses, and computer scientists. The educational booklets were developed to prepare children and adolescents for their visit to family members. Based on Piaget’s developmental stages, three educational booklets were developed and published open source: “The Cave in the Forest” [20] for children aged 6 to 10, “The Journey” [21] for children aged 11 to 13, and “When it’s your turn: A guide for adolescents who have a family member in the ICU” [22] for adolescents from 14 to 17. After the booklets were prepared and printed, we started the research project. The booklets are provided in Supplementary materials.

The research project was conducted in three medical ICUs in northern Italy where children were generally allowed to visit. In these ICUs, a two-hour meeting was conducted with physicians and nurses to explain the MINVITI project, the study purpose and the recruiting procedure. During this meeting, a one-hour informative training session was held to illustrate ‘good practices’ for preparing patients and children before the visit, as well as how to support children during and after the visit [18]. We also provided the booklets that ICU staff could give to parents and children if they expressed the wish to visit the hospitalized family members.

### Participants and procedure

The study had a quasi-experimental design with pre- and post-intervention measurements. In each ICU, healthcare staff recruited children and adolescents: (1) aged between 7 and 17 because, according to Piaget’s theory, children of these age groups are in concrete and formal operational stages, and therefore are better able to understand abstract concepts and comprehend the study questionnaires [23]; (2) able to understand and speak Italian; (3) with a close family member (parent, grandparent, brother/sister) hospitalized in the ICU on mechanical ventilation for > 48 h. This cut-off was determined based on the anesthesiology literature [24], as it helps to exclude patients who are too healthy to stay in the ICU and receive visits, as well as those who are too critically ill to survive ICU hospitalization. Children were excluded from the study if their loved ones were hospitalized because of suicidal attempts or were at risk of immediate death. During the first three days of family members’ hospitalization, both parents or one parent, if the other was the patient, were approached by ICU staff (physician and a nurse or psychologist) after the routine medical update conversation. ICU staff members explained the aims of the project and asked the parents if they wanted their child to participate in the study. Upon parental consent, the parents were provided with a link to an online questionnaire for their children. The questionnaire was accessible through a web link shared with the parent and was completed at home. Parents provided informed consent by accessing the first part of the online questionnaire. If parental consent was not obtained, the questionnaire could not be submitted or completed. The questionnaire was composed of a sociodemographic section and three scales: the separation anxiety sub-scale of the Spence Children’s Anxiety Scale (SCAS) [25] that measures separation anxiety, the Children’s Revised Impact of Event Scale (CRIES-8) [26] to screen for Post-Traumatic Stress Disorder, and the Positive and Negative Affect for Children (PANAS-C) [27] to assess the children’s emotional state.

After completing the pre- questionnaire and if the patient’s situation was stable and (s)he did not present disturbing changes in bodily appearance or behavior, ICU staff and parents discussed whether it would be opportune for the children and adolescents to visit their family members. If parents and children agreed to visit (visitation group), the age-appropriate booklet was offered and a facilitated visit was organized by ICU staff. Visits to family members were not organized if parents or children refused (non-visitation group). Therefore, the non-visitation children never entered the ICU. Only the children of the visitation group entered the ICU. The visit was generally organized within 7–10 days of ICU admission. The visit was prepared by ICU staff (physicians, nurses or psychologists). On the day of the visit, the staff prepared the patient for the visit and used a curtain to ensure privacy and protect children from the sight of other patients. Before the visit, a physician with a nurse or psychologist welcomed the children, explored their understanding of the situation, prepared them for what they would see and what to expect from interacting with the patient, and normalized possible emotional reactions. During the visit, the other parent was present if the child wanted. One ICU staff member remained in the room giving information about the machines, proposing little caring gestures that were possible and was available to answer any questions. Finally, private time between the patient and children was ensured, limiting the duration of the visit according to the comfort of both the child and the patient. After the visit, a short debrief with the child and parent was conducted by asking how the visit had gone and if there were additional questions. One day after the visit, a link to the post- questionnaire was emailed to parents for children and adolescents to complete.

**Fig. 1:**
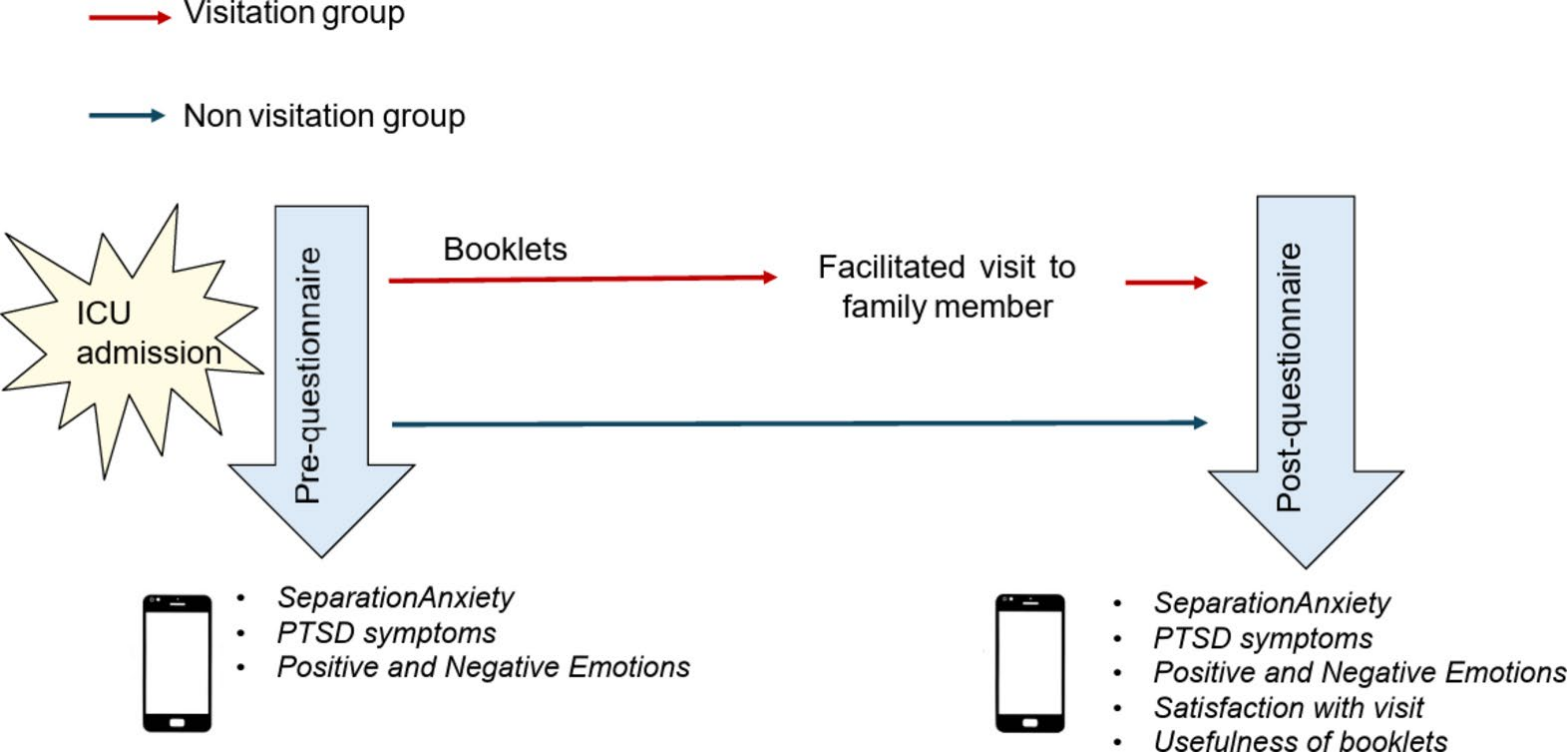
Research design

Parents of the children and adolescents who did not visit received a link to the same questionnaire one week after completing the pre-questionnaire. In case of death of the family member before completion of the post-intervention questionnaire, participation in the study was interrupted. The research design is illustrated in Fig. 1.

## Measurements

### Sociodemographic characteristics

In the pre-intervention questionnaire children were asked to provide information regarding their age, gender, and relationship with hospitalized family members. In the post-intervention questionnaire, information on whether they visited their family member or not, the reason for not doing so, and their satisfaction in the case of visiting was collected.

### Spence children’s anxiety scale (SCAS)

Spence Children’s Anxiety Scale (SCAS) is a self-report questionnaire designed to screen children at risk for anxiety symptoms [25]. The scale consists of six subscales that assess separation anxiety, social phobia, obsessive-compulsive disorder, panic-agoraphobia, generalized anxiety, and fear of physical injury [28]. The SCAS consists of 44 items that assess specific anxiety symptoms related to the six subscales, and others serving as positive filler itemsto reduce negative response bias. In this study, we administered only the separation anxiety subscale of the Italian validated version of SCAS [29]. The separation anxiety subscale comprises 6 items. Children rated the frequency with which they had experienced each item on a 4-point scale ranging from never (scored 0) to always (scored 3). The total subscale scores range from 0 to 18. Lower scores indicate reduced anxious symptomatology. In the analysis, the scale was used as a continuous variable. In this study, the SCAS showed acceptable internal consistency, with a Cronbach’s α of 0.66.

### Children’s revised impact of event scale (CRIES-8)

Children’s Revised Impact of Event Scale (CRIES-8) [30] is a self-report questionnaire designed to screen children at risk for Post-Traumatic Stress Disorder (PTSD). This scale is a reduced 8-item version of the CRIES-13. The 8-item version consists of four questions assessing intrusion and four questions assessing avoidance. The original 13-item version (CRIES-13) includes items of the CRIES-8 and five more questions to assess hyperarousal. In this study, we used the Italian validated version of CRIES-8. Children rated the frequency with which they had experienced each item during the past week using a 4-point Likert-like scale ranging from not at all (scored 0) to often (scored 3). The total score ranges from 0 to 24. Lower scores on each scale indicated a minor risk of developing PTSD. Clinically, if the total score is 17 or more, the probability is very high that a child will be diagnosed with PTSD 26. In the analysis, the scale was used as a continuous variable. In this study, the CRIES-8 showed good internal consistency, with a Cronbach’s α of 0.74.

### Positive and negative affect for children (PANAS-C)

Positive And Negative Affect Scale for Children (PANAS-C) is a self-report questionnaire designed to assess positive and negative emotions in children and adolescents [27]. PANAS-C consists of 30 items that assess specific emotions relating to the Positive Affect and Negative Affect subscales. In this study, we used the Italian validated version of PANAS-C [31], composed of a 12-item Positive Affect Scale and 15-item Negative Affect Scale.

Children and adolescents rated the frequency with which they experienced the feelings described in each item over the past few weeks, using a 5-point Likert-type scale ranging from not at all (scored 0) to often (scored 4). The total score of the Positive Affect Scale ranges from 0 to 48, and the total score of the Negative Affect Scale ranges from 0 to 60. Lower scores on each scale indicate a reduced prevalence of positive or negative emotions. In the analysis, the scale was used as a continuous variable. In this study, the PANAS-C showed good internal consistency, with Cronbach’s α = 0.73.

### Ethics

The research project was approved by the Ethics Committee of the University of Milan (n° 2/20) and was conducted according to the principles stated in the Declaration of Helsinki 1975, as recently amended. Either parents, or a single parent if the other was hospitalized in the ICU, provided informed consent for the participation of their children in the research. The data collected were anonymized.

### Statistical analysis

Descriptive statistics were used to describe sociodemographic data and psychological variables. Given the small number of children and non-normality of some distributions, non-parametric tests were used. Baseline comparison of sociodemographic data (gender, age and relationship with hospitalized family members) and psychological variables of the two groups of children was conducted using Chi square test and Mann-Whitney test. To assess pre and post-intervention differences in psychological well-being in the children who visited and in children who did not visit, Wilcoxon signed-ranked test for paired samples was used for each group. To detect if the changes in psychological variables were significantly different in the two groups of children, the difference (∆) between post and pre-questionnaire scores was computed for each psychological variable. Mann Whitney test for independent samples was then conducted to assess if the differences (∆) in psychological variables of the two groups were significant. Median and interquartile range (IQR) were used to describe the psychological variables. Pearson’s correlation coefficient was used to quantify correlation. Significance level was set at p ˂0.05. All analysis were conducted with SPSS19.

## Results

**Fig. 2:**
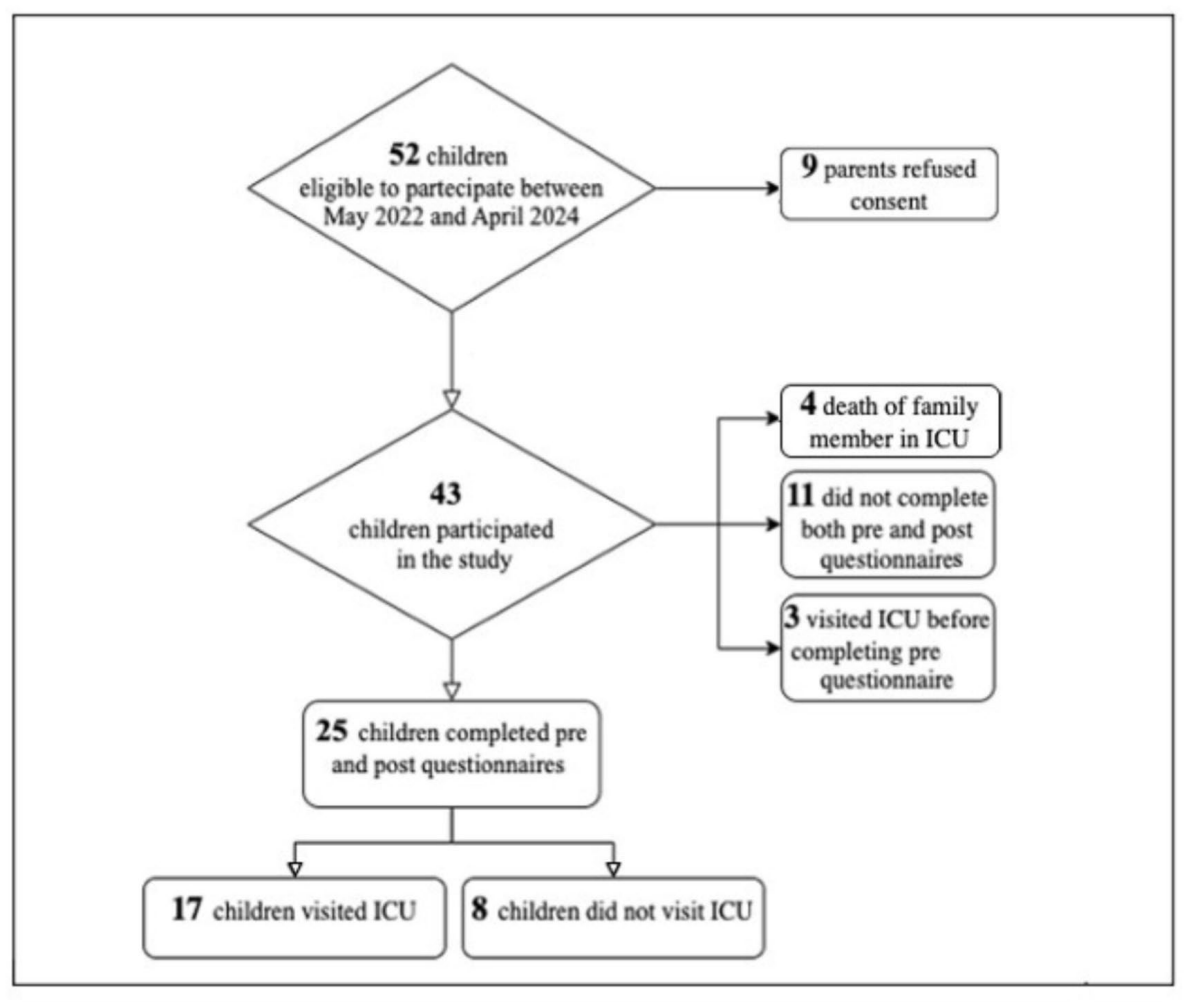
Results of the recruiting process

Of 52 children eligible to participate in the study, 9 refused consent and 43 (83%) agreed to participate. Of these, 4 left the study due to the death of their family member, 3 visited ICU before completing pre-questionnaires and 11 did not complete both pre and post-questionnaires and were therefore excluded from the analysis (Fig. 2).

The sociodemographic data of the 25 children who completed both pre- and post-questionnaires are reported in Table 1.

**Table 1:** Sociodemographic characteristics of children involved in the study

Variable	All children Total *N=25*	Children who visited Total *N=17* (%)	Children who did not visit Total *N=8* (%)
Gender			
Male	13	10 (59)	3 (38)
Female	12	7 (41)	5 (63)
Age (in years)			
Mean (SD)	10.92 (2.69)	11.76 (2.43)	9.13 (2.41)
Family member in ICU			
Sibling	3	2 (12)	1 (13)
Parent	8	7 (41)	1 (13)
Grandparent	14	8 (47)	6 (75)
Hospital			
San Carlo	9	6 (35)	3 (38)
Niguarda	9	4 (23)	5 (63)
Legnano	7	7 (41)	−

Of these 17 (68%) visited their family members and 8 did not. Of the 8 children who did not visit, 4 reported that they did not visit because they were told they could not, 1 because (s)he did not want to, and 3 did not know. Children who visited were older than children who did not visit [11.76 years old (2.43) vs. 9.13 years old (2.41); U = 109.000; *p* = 0.016] and, at pre-questionnaires, presented higher separation anxiety [8 (6; 10.5) vs. 5.5 (5; 7.75); U = 102.500; *p* = 0.043] and lower positive affects [33 (23.5; 36) vs. 47 (36.5-51.75); U = 30.000; *p* = 0.027]. There were no statistical differences in PTSD symptoms (U = 94.000; *p* = 0.129) and negative affects (U = 97.500; *p* =.085) between the two groups of children at the baseline. Children who visited and those who did not visit, did not differ in their relationship with the family member hospitalized (parent or other) in the ICU (Chi^2^ = 2.056; df = 1; *p* = 0.152, Fisher test *p* = 0.205). All children (100%) who visited stated they were satisfied with the visit. Children who visited reported a decrease in separation anxiety, PTSD symptoms and an increase in positive affect, whereas children who did not visit reported a decrease only in PTSD symptoms (Table 2).

**Table 2:** Psychological well-being of children who visited and did not visit

Variable	Children who visited		Wilcoxon signed-ranked test	Children who did not visit		Wilcoxon signed-ranked test
	Median Pre (IQR)	Median Post (IQR)	*p*-value	Median Pre (IQR)	Median Post (IQR)	*p*-value
Separation anxiety	8 (6; 10.5)	5 (4; 8)	0.004	5.5 (5; 7.75)	5.5 (5; 8.25)	0.461
PTSD symptoms	25 (19; 28)	18 (9.5;23.5)	0.008	13 (12; 24.5)	10 (5; 17.50)	0.018
Negative Affect	43 (38; 48.5)	37 (28.5; 44)	0.319	32.5 (28.25; 44)	31.5 (19; 38.75)	0.865
Positive Affect	33 (23.5; 36)	34 (24; 41.5)	0.010	47 (36.5-51.75)	44 (35.5; 51.25)	0.063

Median and IQR of their paired differences are reported in Table 3.

**Table 3:** Difference in psychological well-being of children who visited and who did not

Variable	Children who visited	Children who did not visit	Mann Whitney
	Median Post-Pre (IQR)	Median Post-Pre (IQR)	*p*-value
Separation anxiety	-3 (-5.5; − 0.5)	0 (0; 1)	0.006
PTSD symptoms	-4 (-9.5; -1.5)	-5 (-10.25; -2.5)	0.754
Negative Affect	-5 (-10; 0)	-5.5 (-11.5; 1.5)	0.932
Positive Affect	-1 (-2; 12.5)	-0.5 (-2; 4.5)	0.549

Table 3 shows the comparison of the post-pre differences of children who visited and children who did not. Children who visited their family members reported a greater decrease in separation anxiety [-3(-5.5; -0.5) vs. 0(0; 1); *U* = 22.500; *p* =.006] than children who did not visit. The association between the post-pre difference in separation anxiety and visits remained after adjusting for age (*p* <.05). Furthermore, no correlation was found with age (*p* >.05). Other post-pre differences were not found to be statistically significant (*p* >.05).

**Fig. 3:**
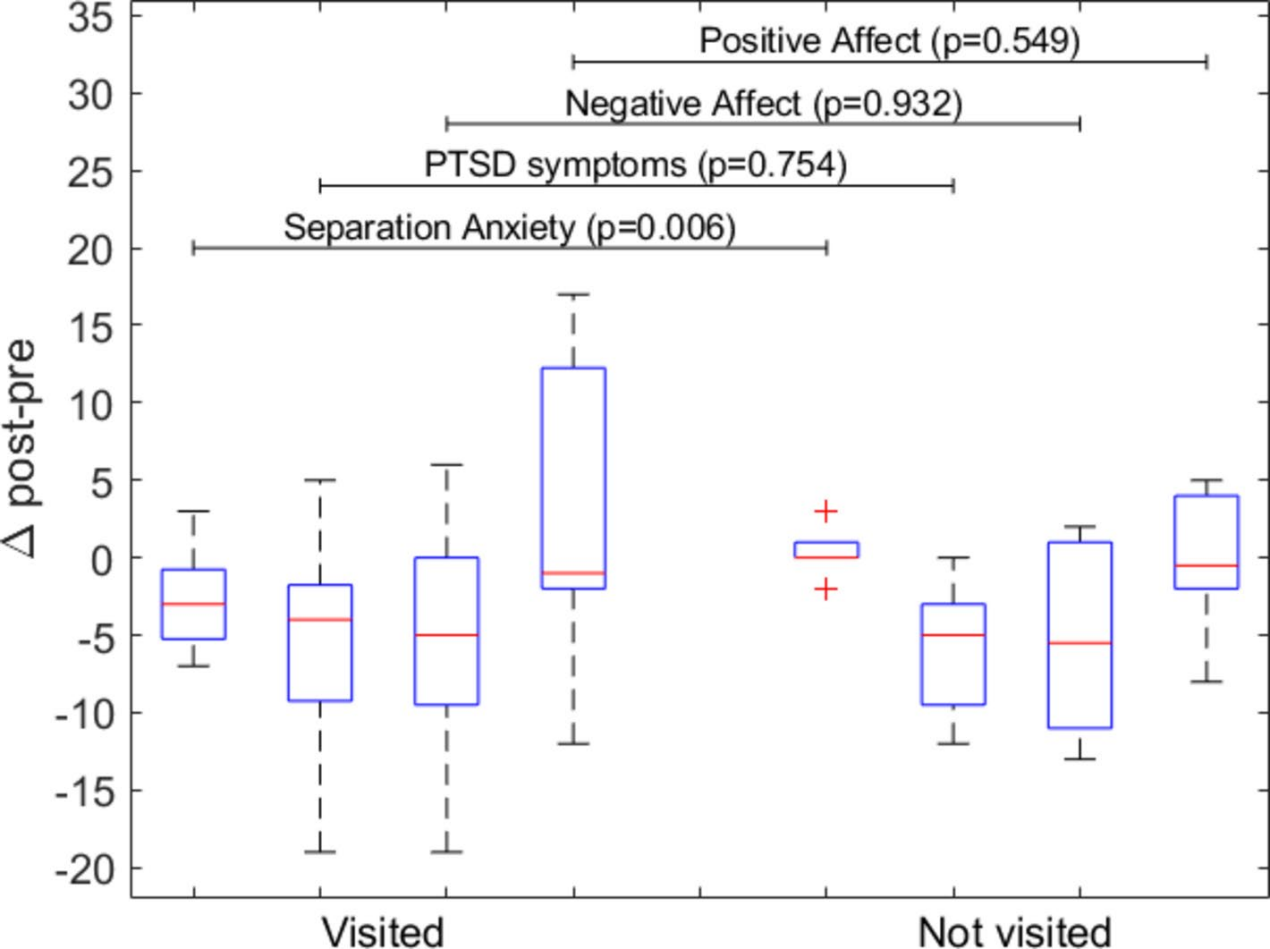
Boxplot of ∆ post-pre values for children who visited and who did not

The boxplot containing median and interquartile range (IQR) of **∆** post-pre values for children who visited and who did not is reported in Fig. 3. P-values are reported for each comparison.

## Discussion

Despite family-centered care principles encouraging children’s visits to family members in adult ICUs, barriers and resistance persist among clinicians and parents [10, 32]. This quantitative quasi-experimental study assessed, through a pre-post measure design, if a facilitated visit to family members in ICU could enhance children and adolescents’ psychological well-being in terms of separation anxiety, PTSD symptoms, and positive and negative affects.

As randomization of children to the visitation or non-visitation group was not possible, baseline differences of children who visited and who did not, were compared. The majority (68%) of children in our sample visited their family members. These children were older and presented higher separation anxiety and lower positive affects at pre-questionnaires than children who did not visit. A possible explanation for these findings is that, although the difference was not significant, in the visitation group, the hospitalized family member was often a parent, whereas in the non-visitation group, it was frequently a grandparent. The relationship between the child and the ICU patient could have influenced baseline levels of separation anxiety and negative affects. Due to the strong attachment bond between parents and children and its importance for children’s emotion regulation, it is possible that when a parent is hospitalized in ICU, children experience greater separation anxiety and more intense negative emotions compared to when another family member is hospitalized [15]. In addition, as the decision to visit or not was left up to parents and children, it is possible that older children were better able to articulate and assertively express their desire to visit than younger children or were perceived by parents and clinicians as less vulnerable. It is also possible that the higher levels of separation anxiety and distress presented by these children served as a motivational drive to speak up and ask to visit their loved ones.

Despite these baseline differences, our results showed that children who visited their family members presented a significant decrease in separation anxiety compared to children who did not visit. The reduction of separation anxiety is important not only for the child’s well-being but also for their emotional and social development. Separation anxiety is a normal response to a family member’s hospitalization but can become problematic if it is persistent and intense. Studies on children and adolescents highlight that high levels of separation anxiety are correlated with sleep difficulties [33] and behavioral problems. Separation anxiety has the potential to negatively impact the child’s social and emotional functioning [34]. Consistently with Bowlby’s attachment theory [15], preserving family relationships and physical closeness during stressful times, such as during an ICU hospitalization, was associated in our study with decreased separation anxiety in children. The results of our study confirm that visiting family members in ICU is not just an ethical practice but a practice that addresses a fundamental psychological need for children, the need for attachment [16].

Contrary to our hypothesis, children who visited did not show a greater reduction in PTSD symptoms compared to children who did not visit. However, more importantly, nor did they show a significant increase in PTSD symptoms. Some studies [31] pointed out that one of the main barriers for not allowing children in the ICU is the fear of parents and clinicians that they could be traumatized by the ICU environment, the sight of other patients and the sight of their loved ones in a critical condition. Consistently with the findings of other studies on adult family members [35], our findings show that PTSD symptoms are present among children and adolescents who have a family member in the ICU. However, the presence of PTSD symptoms is not necessarily caused or worsened by the visit to the ICU, especially if this is prepared and facilitated, but may be due to the uncertain and severe medical condition that affects their loved ones and that traumatically altered their everyday life and family functioning. Having found that children who visited did not show a significant increase in PTSD symptoms compared to children who did not visit is important, since it highlights that not allowing children to visit to protect them from possible traumatic aspects is not based on empirical evidence. On the contrary, other studies showed that the proximity to the family member during the visit allowed children to gain a better understanding of the situation [17] and correct scary fantasies that could be generated from not seeing them [36]. Additionally, it is worth noting that, at baseline and therefore before even entering the ICU, children who visited their loved ones presented a median PTSD score that was above the clinical threshold, whereas the children in the non-visitation group had a median PTSD score below the clinical threshold. Although this difference in PTSD scores was not statistically significant, it is clinically relevant and may be attributed to the fact that children who visited often had a parent hospitalized in the ICU. The high PTSD symptoms at the baseline may reflect the traumatic experience of having a parent hospitalized, along with the resulting disruption of routine and emotional security. Children and adolescents with a parent hospitalized in ICU should therefore receive special clinical attention due to the underling traumatic experience. Having access to a psychological counseling service for the families of patients in the ICU could be helpful in managing the traumatic aspects related to the experience of having a parent hospitalized [37].

In our study, the visit was prepared by using educational booklets and was facilitated by previously trained ICU staff members. All the children who visited were satisfied with the experience, and 16 out of 17 considered the educational booklets useful. This finding is consistent with the study of Ferge et al. [38] who found that adolescents between 12 and 17 years old who visited the ICU were less likely to report feelings of regret than those who did not visit the ICU (2% vs. 9%, *p* =.01). Our findings suggest that educational booklets are a precious resource that can be used to facilitate open communication between children and parents about what happened and prepare children to visit their loved ones. Besides those developed for this study, the first ever published in Italian, several English booklets are available having the same purposes [39, 40, 41]. These booklets could be incorporated into daily clinical practice to prepare children.

Our study has several limitations. It is worth mentioning that our study design did not allow assessment of the impact of the visit without preparation and the use of booklets. This, however, was not considered the main objective of the study. The research design was quasi-experimental because it was not possible or ethical to randomly allocate children to the visitation or non-visitation group. Moreover, the parents and children who decided not to visit were fewer than those who decided to visit, consequently the non-visitation group was less numerous than the experimental group. Additionally, as children in the intervention group had consented to visit their family members, there is a potential for selection bias. This is confirmed by the baseline differences between the two groups. Finally, children’s recruitment was challenging, and we had a high drop-out rate during the study because of the difficult circumstances the families were going through.

Despite these limitations, our results have several practical implications. First, children’s visits have the potential to decrease separation anxiety without causing harm, if they are well prepared. Children should receive age-appropriate information and explanations about the environment they will see in order to be ready for the visit. Clinicians’ explanations and information are important to facilitate children’s understanding of the clinical situation [14, 19]. Educational booklets could be used to explain the condition of their family member. ICU wards need to be prepared to limit the view of other patients and machinery that might cause fear. Furthermore, ICU physicians and nurses should receive training to fully comprehend the needs of children and be prepared to manage their presence in adult ICUs based on recent recommendations [18].

Our study and that of Nicholson et al. [19] found that visiting had a positive psychological impact on children’s separation anxiety, hence strengthening the evidence supporting children’s visits in ICUs. These data should fuel the process of cultural and policy change. To tackle other possible barriers to children’s visits, future studies should provide further data to comprehend the perception of the visit, not only by children but also by parents and inpatients. The illness of a family member is an event that causes major changes in the family unit, involving each member and impacting the family equilibrium. It is therefore important to consider each member’s perspective during hospitalization.

## Conclusions

Family-centered ICU care is nowadays an approach of essential importance [42]. Our findings provide initial evidence supporting policies regarding children’s and adolescents’ visits in adult ICUs. When the right conditions are in place, allowing children to visit in a supportive manner may be beneficial to alleviate their separation anxiety without causing any evident harm. By implementing prepared and supported visits, clinicians can help mitigate the separation anxiety that often affects children when a family member is hospitalized due to a severe medical condition.

## Electronic supplementary material

Below is the link to the electronic supplementary material.

Supplementary Material 1.

Supplementary Material 2.

Supplementary Material 3.

## Data Availability

The participants of this study did not give written consent for their childrens’ data to be shared publicly, so due to the sensitive nature of the research, supporting data is not available. Data will be made available only to Reviewers and Editorial staff upon reasonable request.
