# Supplementary material for: Visiting a loved one in the ICU with the aid of dedicated booklets is associated with reduced separation anxiety in children and adolescents
Source: Child Adolesc Psychiatry Ment Health. 2025 Jun 6;19:65. doi: 10.1186/s13034-025-00906-4 (PMC12142882; doi:10.1186/s13034-025-00906-4)
Supplement: Supplementary file 1 — Supplementary Material 1. [file 13034_2025_906_MOESM1_ESM.pdf]

La caverna nel bosco

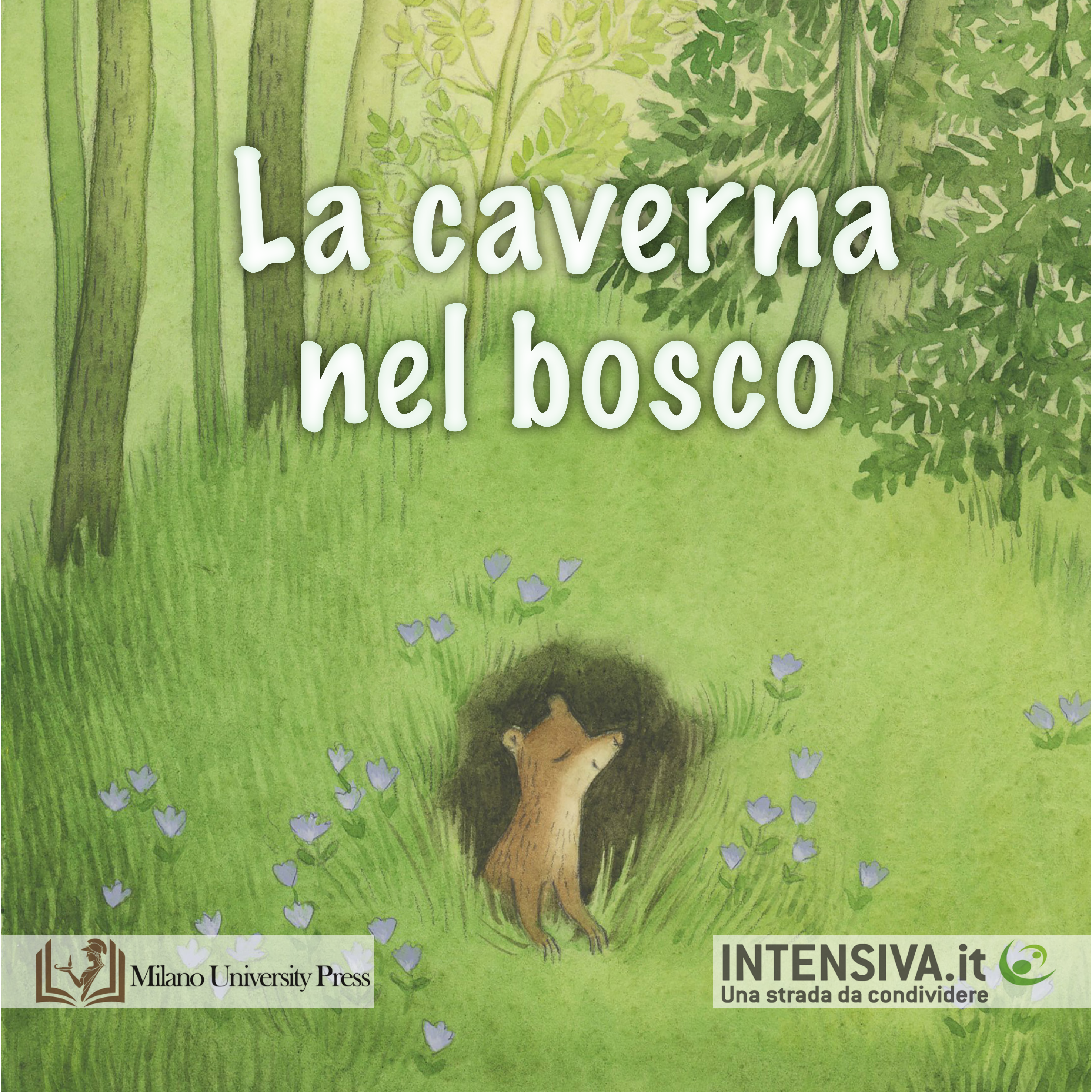

Milano University Press

INTENSIVA.it
Una strada da condividere

La caverna nel bosco

Milano University Press

La caverna nel bosco / di Giulia Lamiani e Michela Maxia. Milano: Milano University Press, 2021.

ISBN 979-12-80325-34-1 (print)

ISBN 979-12-80325-38-9 (PDF)

DOI 10.54103/milanoup.61

Le edizioni digitali dell'opera sono rilasciate con licenza Creative Commons Attribution 4.0 - CC-BY-NC-SA, il cui testo integrale è disponibile all'URL: https://creativecommons.org/licenses/by-nc-sa/4.0/.

Le edizioni digitali online sono pubblicate in Open Access su: https://libri.unimi.it/index.php/milanoup.

© 2021 Giulia Lamiani, Michela Maxia,
© Milano University Press per la presente edizione
Illustrazioni di Giovanni Manna

Pubblicato da:
Milano University Press

Via Festa del Perdono 7 – 20122 Milano

Sito web: https://milanoup.unimi.it

e-mail: redazione.milanoup@unimi.it

L'edizione cartacea del volume può essere richiesta sul sito di SIAARTI (https://www.siaarti.it/).

Questo libro è pensato per tutte quelle famiglie che stanno attraversando un momento difficile perché hanno un famigliare in Terapia Intensiva. In questi momenti è facile chiudersi, ognuno nelle proprie paure o nel proprio dolore. Una storia, letta insieme, potrebbe essere un modo per dar voce ad alcune emozioni, prendersi del tempo per condividerle e sentirsi meno soli.

Potete usare le schede finali di questo libro per aiutare i bambini ad esprimersi e a prepararsi ad un eventuale ingresso in Terapia Intensiva accompagnati da voi e dagli operatori sanitari.

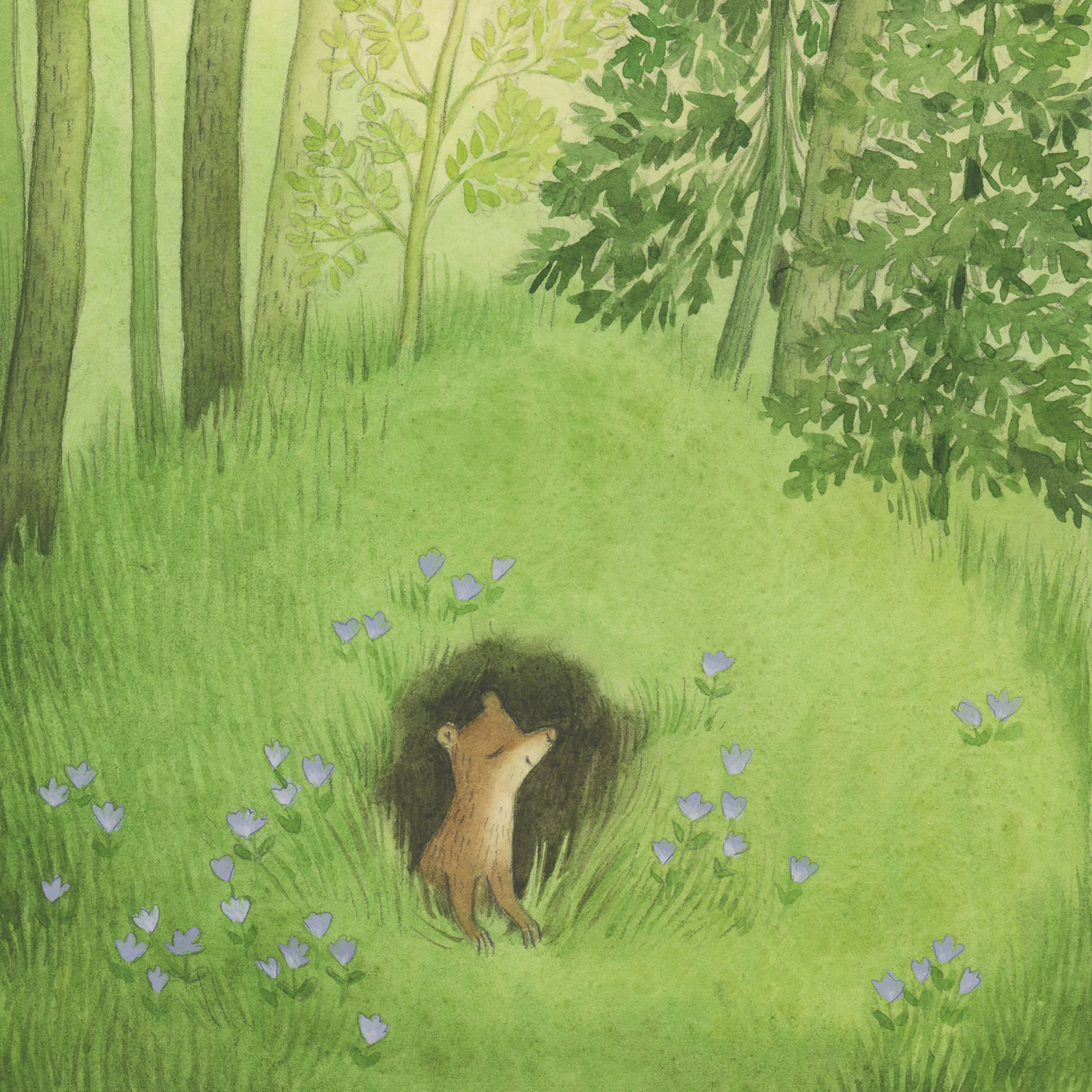

Come tutti gli anni nel grande bosco era finalmente arrivata la primavera. Dagli alberi iniziavano a nascere le foglie nuove, gli orsi tornavano a mettere i musi fuori dalle loro tane e le api andavano in giro in cerca dei fiori più profumati. Quel giorno Leo, un orsetto curioso e vivace, si svegliò di buon umore. Dopo la mattinata di esplorazioni con Tina, un'intrepida orsetta che abitava non lontano, sarebbe andato con mamma Dorotea in cerca di miele. Il letargo era stato lungo e aveva una gran fame!

Si lavò gli occhi e uscì dalla tana alla ricerca di Tina. Leo e Tina si divertivano sempre molto a rincorrere le api e a imparare dagli orsi più grandi a riconoscere gli alberi dove si nascondevano gli alveari. La mamma li avrebbe raggiunti più tardi nella radura del bosco, dopo aver cercato germogli e insetti con papà Gustavo. Leo e Tina arrivarono alla radura del bosco dove ogni anno si davano appuntamento per partire per la grande caccia… Il miele più buono dell'anno li stava aspettando!

Ed eccoli lì nella radura. Il sole era già alto nel cielo ma Dorotea non si vedeva arrivare. Leo iniziò a preoccuparsi e a chiedersi come mai la mamma non fosse lì con loro. Gli sembrava molto strano: a Dorotea piaceva tanto andare a caccia del primo miele e non se la sarebbe persa per nulla al mondo. Poi senza la sua guida come l'avrebbero trovato?!

Ad un certo punto Leo vide arrivare papà Gustavo accompagnato da zia Lisa. Parlavano tra di loro fitto fitto. Leo non capiva bene cosa si stessero dicendo ma dalle loro facce aveva intuito che era successo qualcosa di brutto. Nessuno però gli parlava! E più nessuno gli parlava più si sentiva spaventato e arrabbiato. Perché mai non lo consideravano? Forse non lo vedevano? "Papà cosa è successo?" chiese Leo con un filo di voce "Perché la mamma non è qui con noi?". Papà sapeva sempre tutto e, anche se quel giorno era stranamente sfuggente, Leo era sicuro che gli avrebbe dato una valida spiegazione.

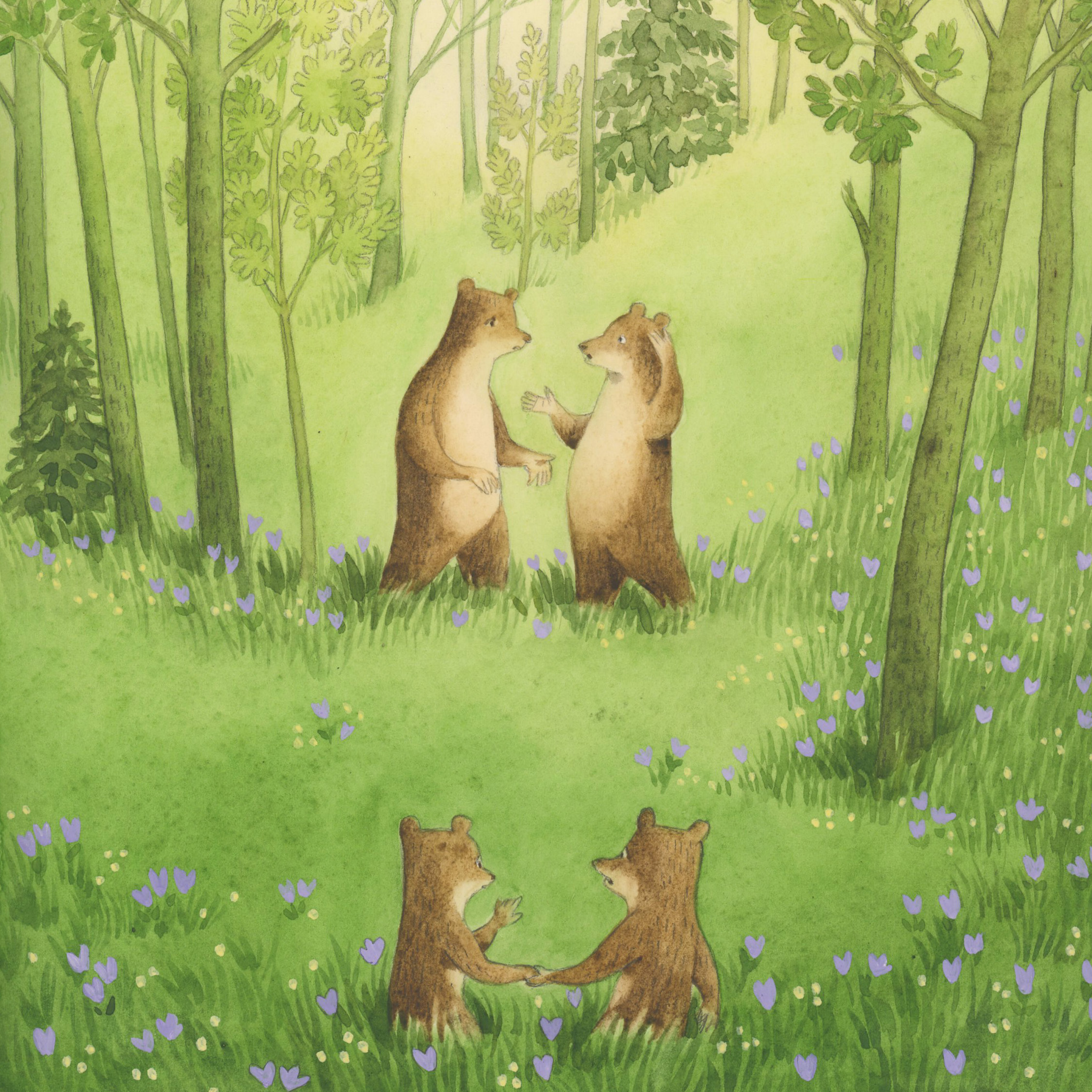

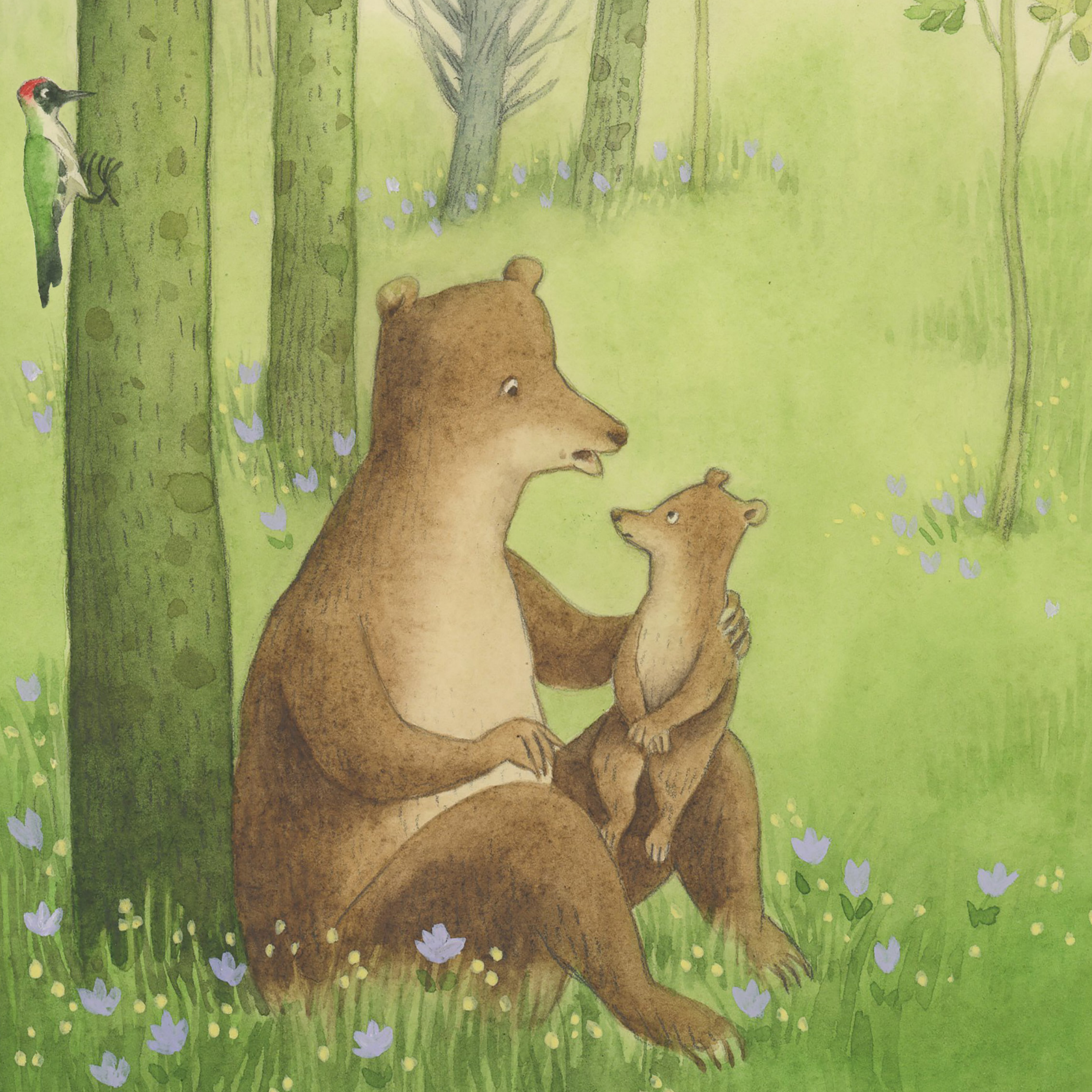

«Leo» gli rispose papà Gustavo «la mamma oggi non potrà venire a cacciare il miele con te. Purtroppo questa mattina, mentre cercavamo germogli e insetti, è scivolata da una roccia e si è ferita gravemente cadendo sul fondo del dirupo. L'abbiamo portata nella caverna del bosco, che pochi conoscono, dove la saggia tartaruga e gli altri animali che custodiscono i segreti della cura si stanno occupando di lei».

A Leo iniziò a ingarbugliarsi lo stomaco, sentì il suo pelo farsi ispido e la paura diventare più grande. Come era possibile che proprio la sua mamma fosse caduta? Gli sembrava tutto così strano. «Papà» disse «forse è colpa mia! Stamattina ho detto alla mamma che avevo tanta fame e lei è andata in cerca di cibo per me!». A quelle parole papà Gustavo fece un sorriso dolce: «Io e la mamma andiamo sempre dopo il letargo a cercare germogli e insetti. Non c'è niente che tu abbia detto o fatto che possa aver causato questo brutto incidente».

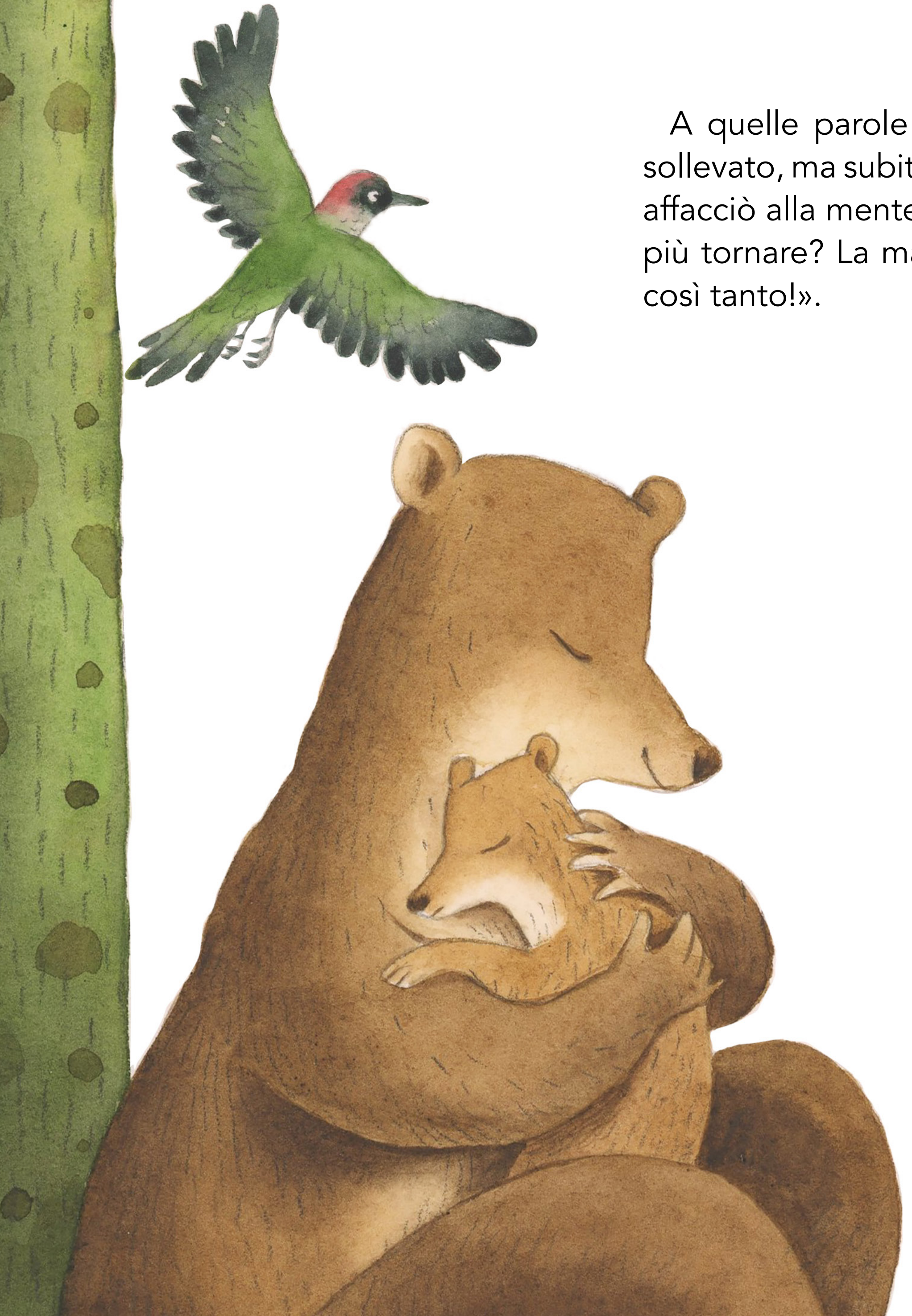

A quelle parole Leo si sentì un po' sollevato, ma subito un'altra paura gli si affacciò alla mente: «E se non dovesse più tornare? La mamma mi manca già così tanto!».

Papà Gustavo strinse forte a sé il piccolo Leo e gli disse: «La mamma manca tanto anche a me, ma è un’orsa forte. Sono sicuro che ce la sta mettendo tutta per guarire e tornare nella tana con noi. Anche gli animali del bosco la stanno curando con tutte le arti che conoscono: i ricci le portano le bacche più succose, i castori hanno costruito i loro famosi boccagli per aiutarla a respirare meglio e gli uccellini le portano l’acqua fresca dell’alta fonte. La mamma ora dorme e non sente male. È come fosse in letargo».

«E io? Che cosa posso fare?» chiese Leo, sentendosi un po’ inutile e sconsolato: «Vorrei tanto aiutarla anche io». «La mamma sa che le vuoi bene» rispose papà Gustavo «e sono sicuro che sorriderebbe se tu, mentre la aspettiamo, continuassi a fare le cose di sempre: esplorare il bosco con Tina, andare a caccia del miele e perfino entrare nella tana con le zampe sporche di fango facendomi arrabbiare! Presto andrò a trovarla e, se ti fa piacere, le dirò che la pensi tanto». Il giorno dopo Gustavo si preparò di buon mattino per andare a trovare Dorotea. Il viaggio non sarebbe stato facile. La caverna del bosco era lontana un giorno di cammino.

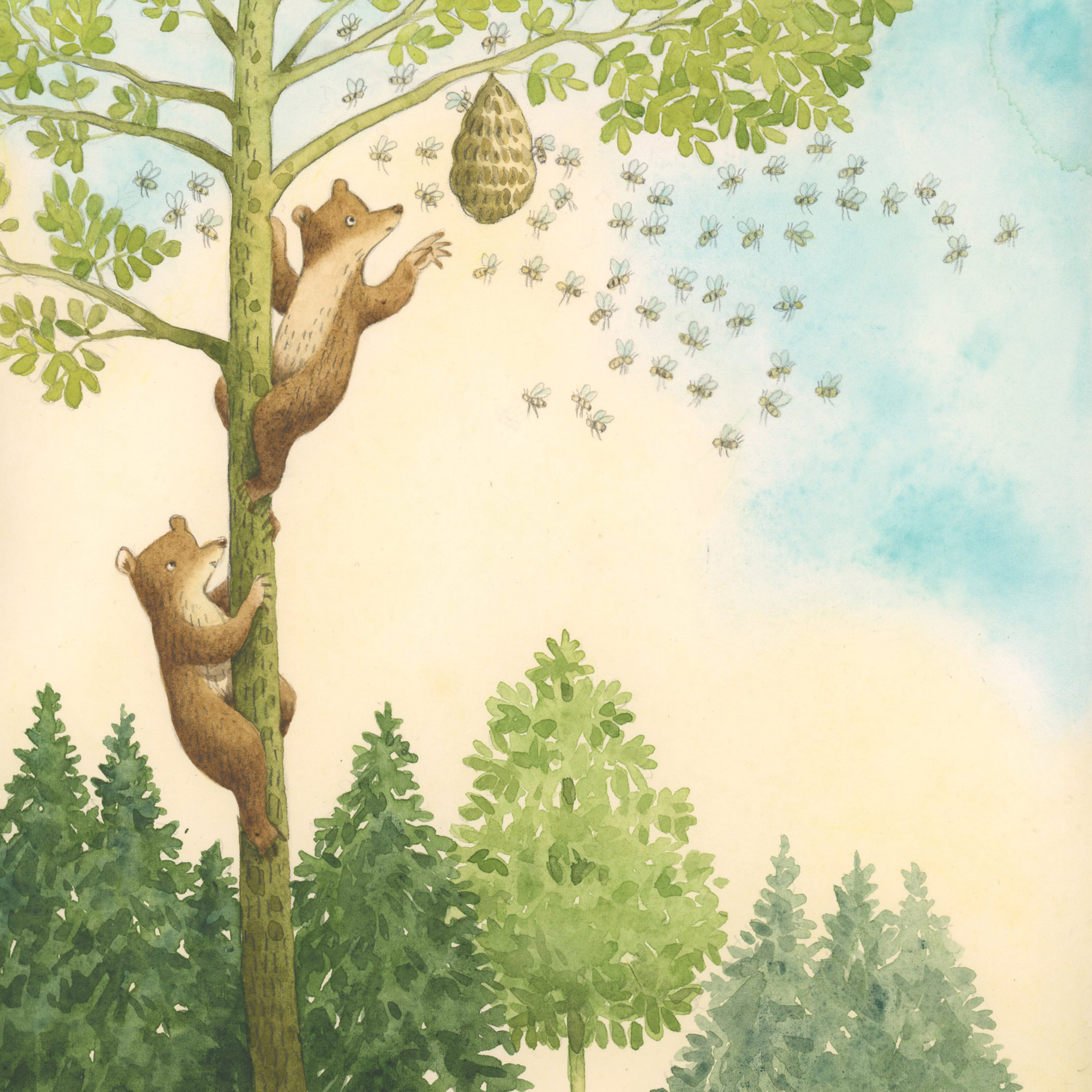

Da quel giorno, papà Gustavo e qualche volta anche zia Lisa si erano più volte messi in viaggio verso la caverna per andare a trovare Dorotea. Leo li osservava e qualche volta ascoltava pezzi dei loro racconti. Come gli mancava la sua mamma! A volte sentiva tanta nostalgia, altre volte si sentiva un po' inquieto. E allora andava a ripararsi tra le braccia del papà. Un po' controvoglia, aveva ripreso a fare le esplorazioni con Tina anche sotto le prime piogge di primavera e, come al solito, era entrato nella tana con le zampe tutte sporche di fango, facendo andare il papà su tutte le furie. Aveva anche provato ad andare a caccia di miele con zia Lisa, ma aveva sempre in mente la mamma. Finché un giorno a Leo venne un'idea. «Papà» disse «voglio venire anche io a trovare la mamma. Credo di essere grande e forte abbastanza per poter affrontare il viaggio. Ho raccolto il primo miele con la zia e vorrei tanto portarglielo!». Papà Gustavo abbracciò forte Leo e gli disse: «Il viaggio verso la caverna è lungo e faticoso, ma se vuoi andare ti accompagnerò. E ricorda che in qualunque momento vorrai tornare indietro, lo potremo fare».

E così il giorno dopo Leo e il papà si incamminarono nel bosco con un favo di miele da portare alla mamma.

Quando arrivarono alla caverna, Leo era molto stanco e agitato, la sua pancia era un groviglio di emozioni e il suo pelo era molto ispido. La caverna era illuminata da un raggio di sole e Leo riuscì a intravedere la sua mamma sdraiata su un letto di foglie. Intorno sentiva i suoni degli animali indaffarati a prendersi cura di lei.

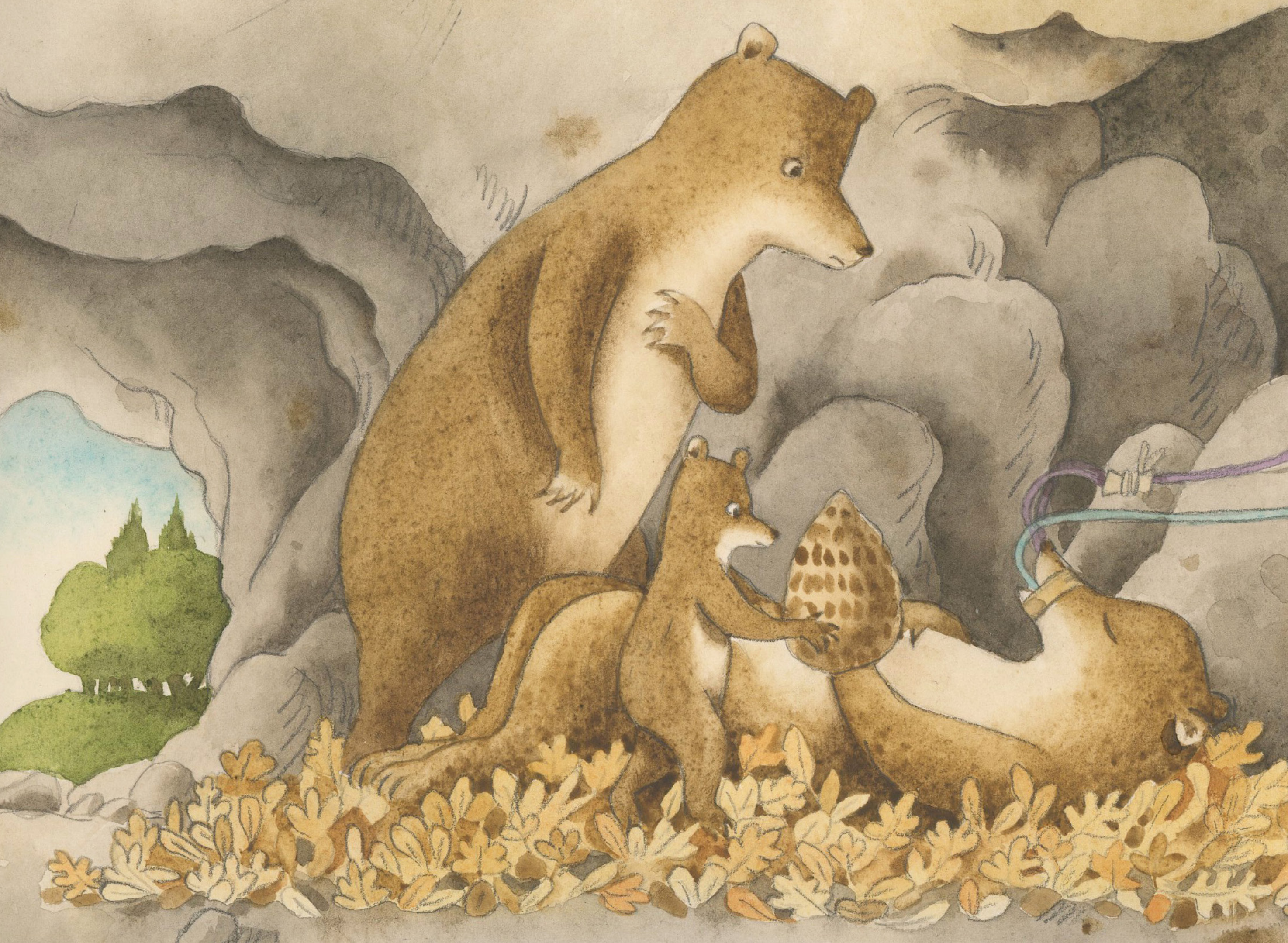

Strinse più forte la mano di papà Gustavo e si avvicinò sempre di più: anche se dormiva e aveva il muso un po' strano, l'orsa sdraiata nella caverna aveva proprio il pelo lucente della sua mamma! Allora Leo, con le zampe un po' tremanti, prese il favo che aveva raccolto e disse: «Mamma, questo è per te!». Papà Gustavo accarezzò Leo sussurrando: «Sei un orsetto davvero coraggioso!».

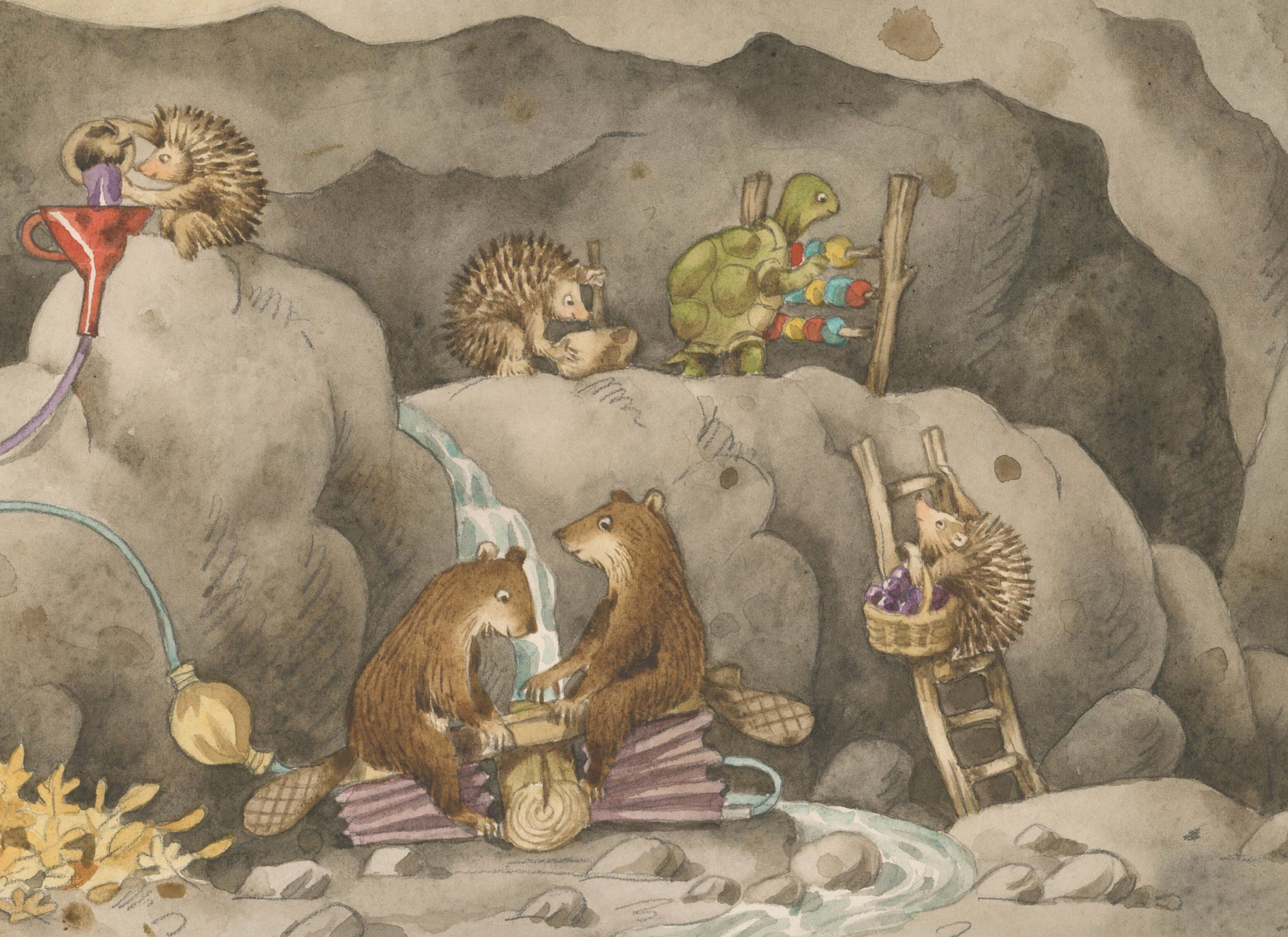

E così, un po’ stanchi e un po’ felici, un po’ tristi e un po’ orgogliosi, i due orsi si incamminarono sulla via del ritorno.

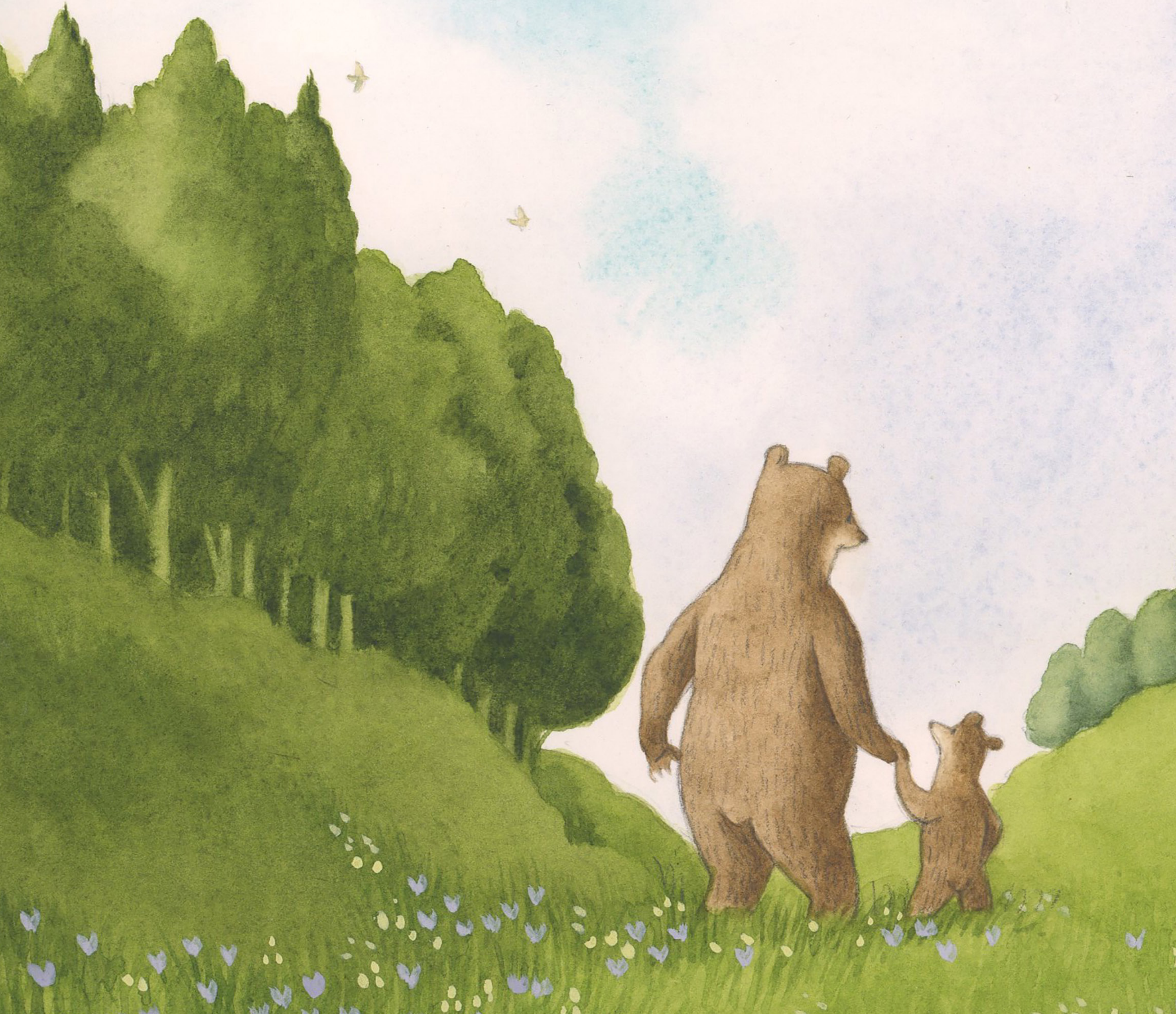

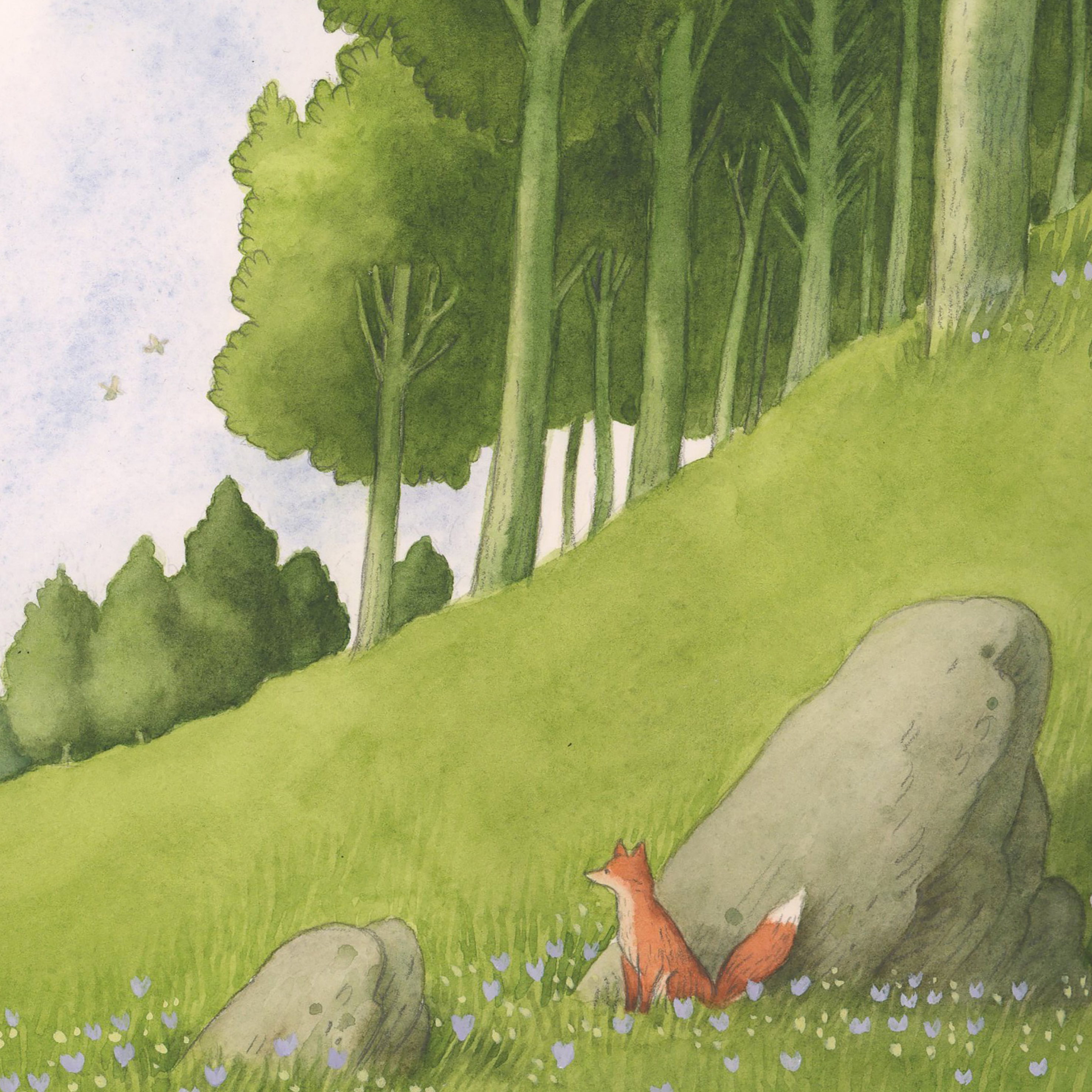

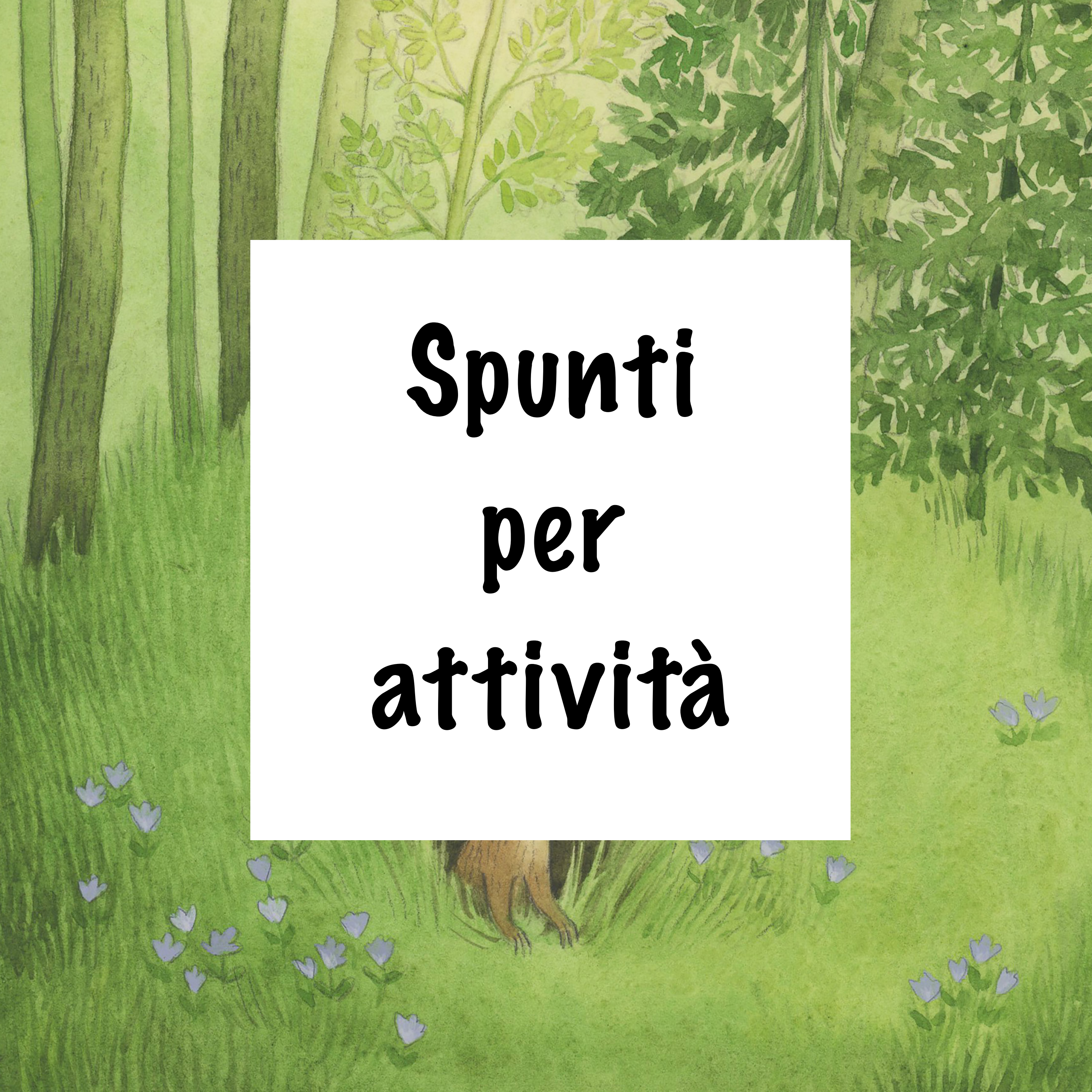

Spunti per attività

1. **CHI SONO.** Nella storia, Leo è un orsetto curioso e vivace. Tu come ti vedi? Se ti va, disegnati qui sotto e scrivi nei palloncini le tue qualità o le cose che ti piace fare!

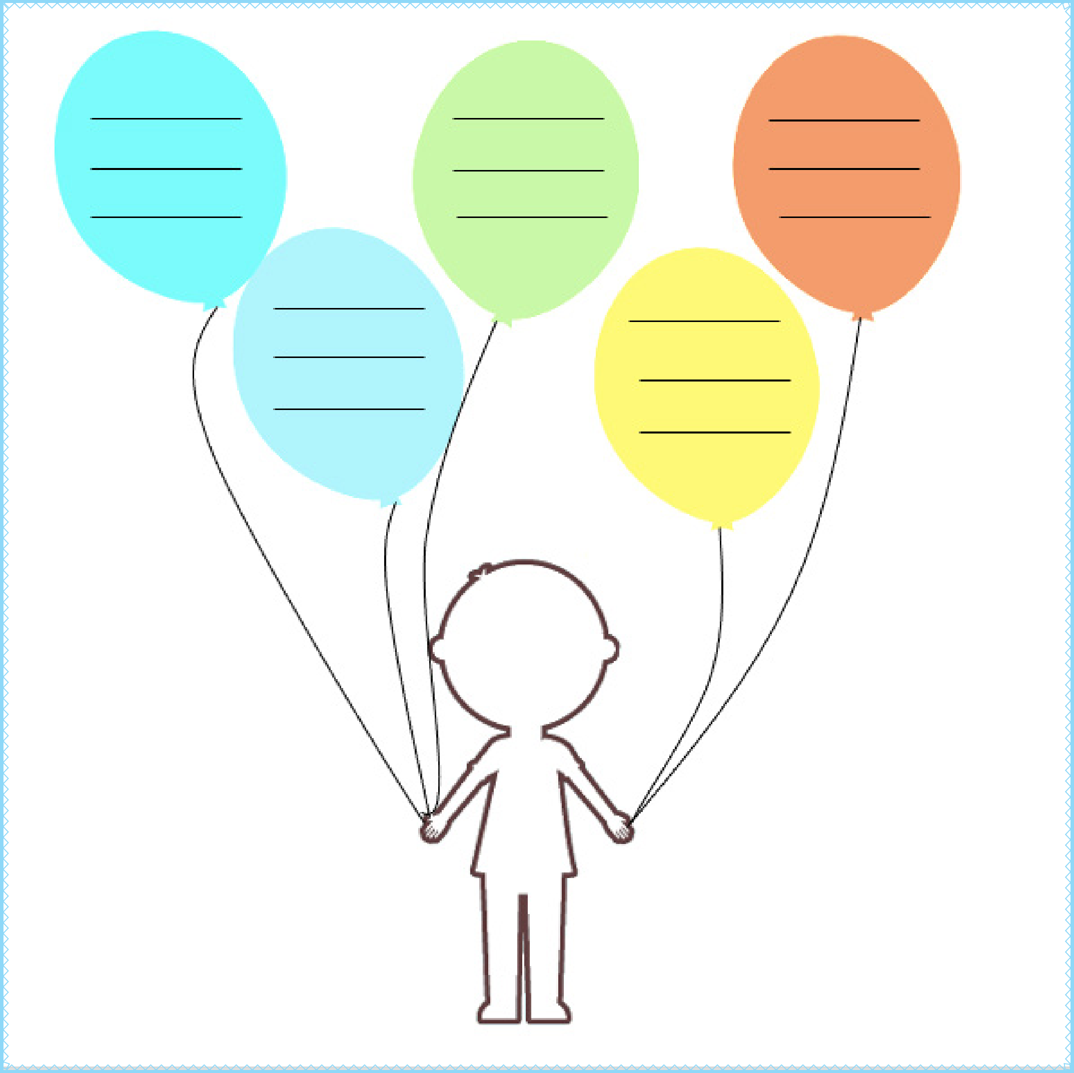

2. **DOVE STO BENE.** Leo ama il suo grande bosco. Tu hai un posto preferito in cui ti piace fermarti a giocare, pensare o riposare? Disegnalo o descrivilo qui sotto.

3. LE MIE PERSONE IMPORTANTI. Leo voleva bene alla mamma, al papà, alla zia e a Tina. Chi sono le persone (o anche gli animali!) importanti per te? Usa lo spazio qui sotto per rappresentarle (puoi disegnare, scrivere, incollare un'immagine o qualcos'altro che te le ricorda).

4. LA TERAPIA INTENSIVA. Mamma Dorotea è curata nella caverna del bosco, accudita dagli animali. Questa sotto è la stanza della **Terapia Intensiva** dove vengono curate le persone. Ci sono tanti strumenti che le aiutano e tanti professionisti che si occupano di loro.

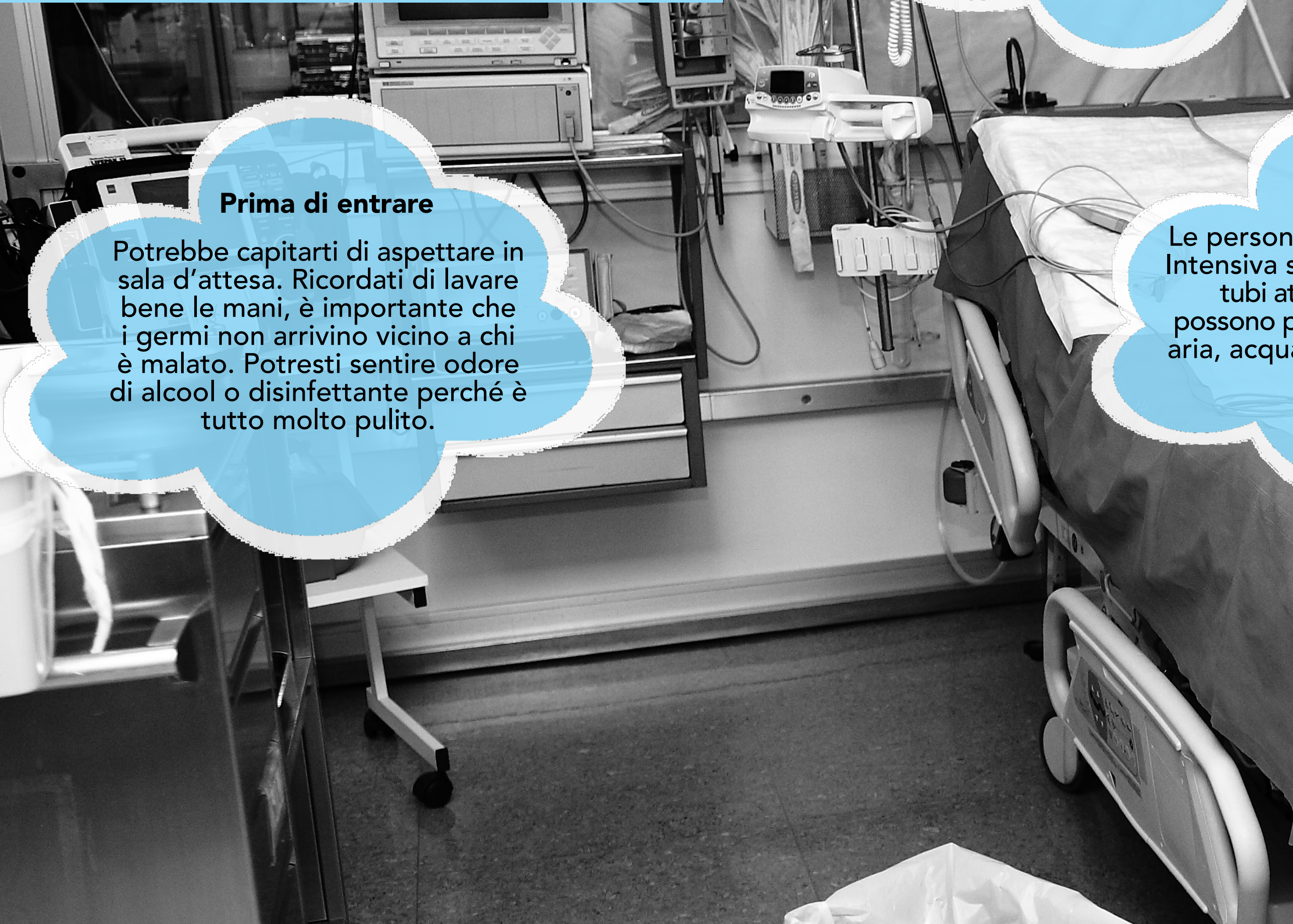

Flebo

Qui si attacca una sacca ch
contiene farmaci e sostanz
nutrienti sciolti nell'acqua c
arrivano al paziente attraver
un tubicino.

Prima di entrare

Potrebbe capitarti di aspettare in sala d'attesa. Ricordati di lavare bene le mani, è importante che i germi non arrivino vicino a chi è malato. Potresti sentire odore di alcool o disinfettante perché è tutto molto pulito.

Le person
Intensiva s
tubi at
possono p
aria, acqua

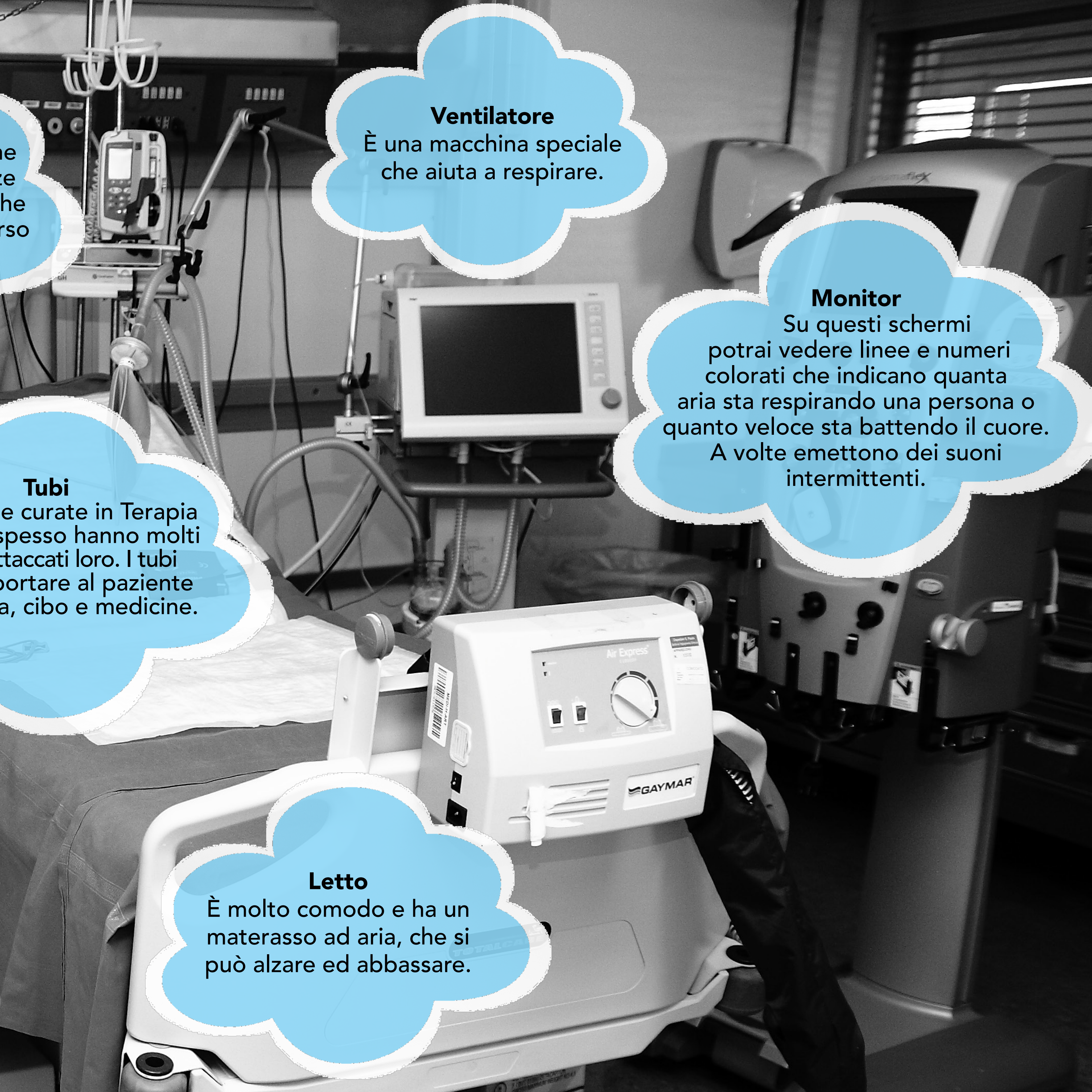

Ventilatore
È una macchina speciale che aiuta a respirare.
Monitor
Su questi schermi potrai vedere linee e numeri colorati che indicano quanta aria sta respirando una persona o quanto veloce sta battendo il cuore. A volte emettono dei suoni intermittenti.
Tubi
e curate in Terapia spesso hanno molti taccati loro. I tubi portare al paziente a, cibo e medicine.
Letto
È molto comodo e ha un materasso ad aria, che si può alzare ed abbassare.
Air Express
GAYMAR

5. CHE COSA POSSO FARE

Leo nella storia decide di portare a sua mamma il miele. Qui sotto c'è una lista di cose che potresti fare se vai in Terapia Intensiva a visitare la tua persona cara. Completa la lista con le cose che ti piacerebbe fare per lui/lei e chiedi ad un adulto di aiutarti a metterle in pratica!

- Farle un disegno o scrivere un bigliettino.
- Tenere le sue mani e raccontare le cose che hai fatto.
- Farle ascoltare una canzone che vi piace.
- Portarle una foto di te e della tua famiglia da attaccare vicino al suo letto.
- ______________________________
- ______________________________
- ______________________________
- ______________________________

Questo libro è rivolto ai bambini dai 6 ai 10 anni che hanno una persona cara ricoverata in Terapia Intensiva. Tutti noi (medici, infermieri, psicologi...) desideriamo essere d'aiuto perché – come familiari di malati gravi – non vi sentiate soli in questo momento di legittima preoccupazione. Il nostro impegno si unisce a quello di tanti altri reparti di Terapia Intensiva, in Italia e nel mondo, per "umanizzare" le cure offerte ai malati e ai loro familiari. Sul sito www.intensiva.it potrete accorgervi che non siete soli: troverete racconti, riflessioni, spiegazioni oltre ad informazioni specifiche sul reparto di Terapia Intensiva in cui è ricoverato il vostro caro.

Per ulteriori domande: info@intensiva.it

Questo libro fa parte di un più ampio progetto di ricerca-azione (Progetto MinViTI) avente l'obiettivo di migliorare il benessere psicologico dei minori che hanno un familiare ricoverato in Terapia Intensiva. **Il progetto è stato finanziato attraverso un Seal of Excellence (SoE) dal Piano di Sostegno alla Ricerca (2020) dell'Università degli Studi di Milano.** Il libro è promosso in collaborazione con il Progetto Intensiva.it, un'iniziativa scientifica supportata da SIAARTI, Aniarti, AAROI-EMAC, dedicata all'umanizzazione delle cure in Terapia Intensiva.

Testi: **Giulia Lamiani** e **Michela Maxia**

Illustrazioni: **Giovanni Manna**

Sezione Psicoeducativa a cura di: **Michela Maxia, Giulia Lamiani, Federica Bonazza**

Gruppo di lavoro: **Giovanni Mistraletti, Massimo Walter Rivolta, Federica Bonazza, Elena Vegni**

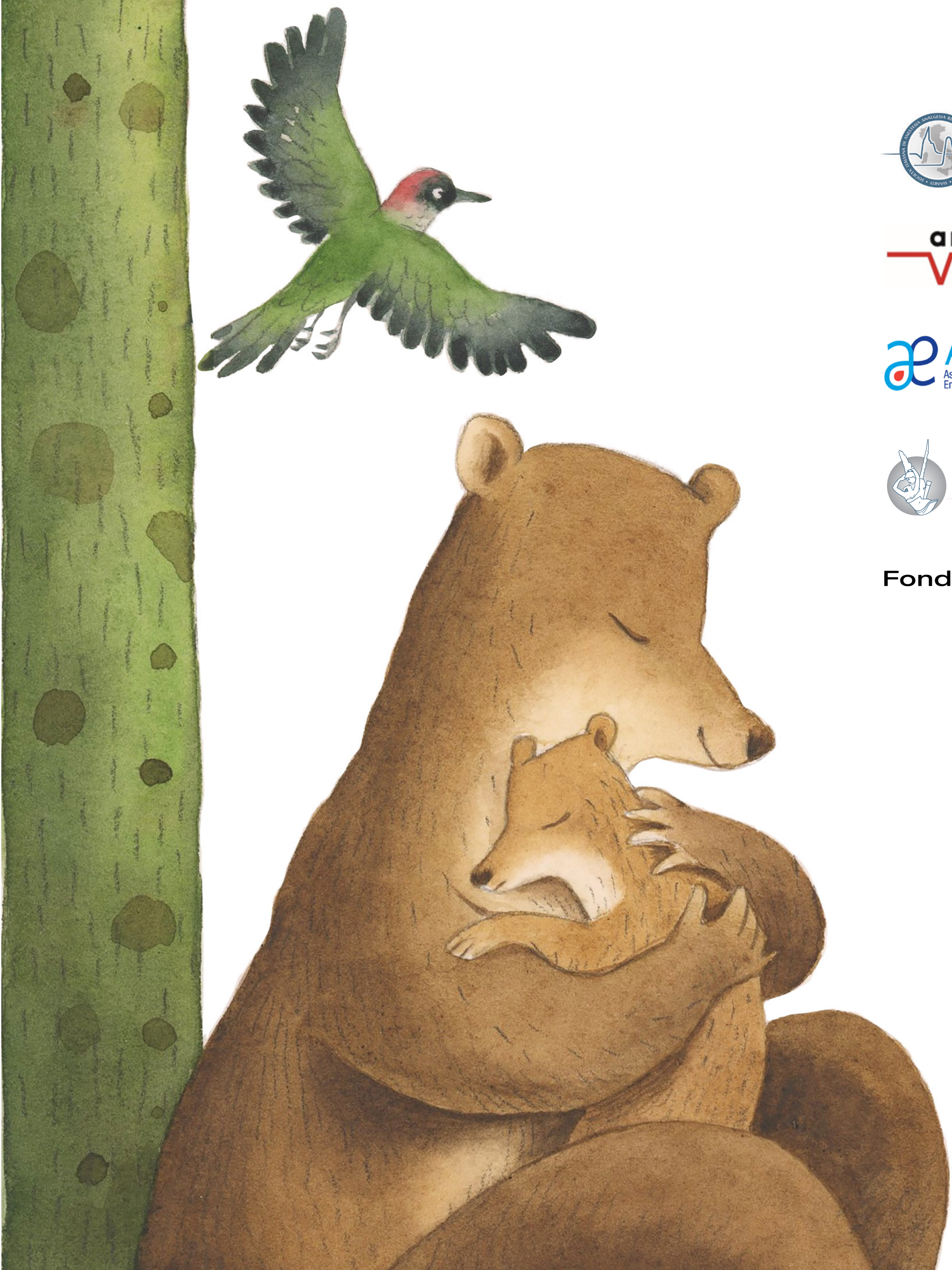

SIAARTI
PRO VITA CONTRA DOLOREM SEMPER

aniarti
associazione nazionale infermieri di area critica

AAROI EMAC
Associazione Anestesisti Rianimatori Ospedalieri Italiani
Emergenza Area Critica

HEROIC bundle
HUMANIZATION TO ENHANCE RECOVERY ON INTENSIVE CARE

Fondazione Štěpán Zavřel
