# Supplementary material for: Visiting a loved one in the ICU with the aid of dedicated booklets is associated with reduced separation anxiety in children and adolescents
Source: Child Adolesc Psychiatry Ment Health. 2025 Jun 6;19:65. doi: 10.1186/s13034-025-00906-4 (PMC12142882; doi:10.1186/s13034-025-00906-4)
Supplement: Supplementary file 2 — Supplementary Material 2. [file 13034_2025_906_MOESM2_ESM.pdf]

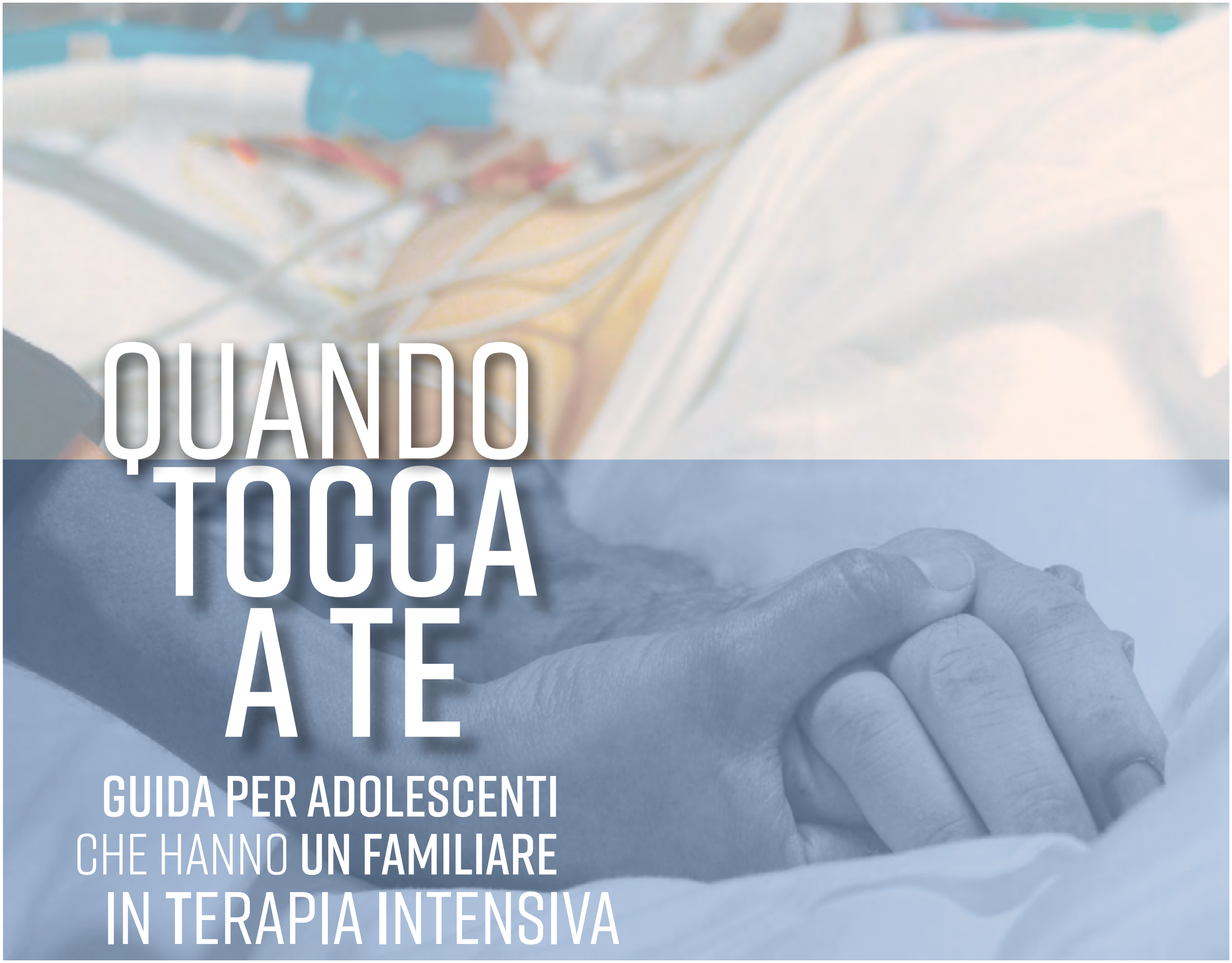

QUANDO TOCCA A TE

GUIDA PER ADOLESCENTI CHE HANNO UN FAMILIARE IN TERAPIA INTENSIVA

Milano University Press

INTENSIVA.it
Una strada da condividere

Quando tocca a te:
guida per adolescenti che hanno
un familiare in Terapia Intensiva
di Giulia Lamiani, Michela Maxia, Stefano Iodice, Giovanni Mistraletti.

DOI 10.54103/intensiva.135

Le edizioni digitali dell'opera sono rilasciate con licenza Creative Commons Attribution CC-BY-SA 4.0,
il cui testo è disponibile all'URL: https://creativecommons.org/licenses/CC-BY-SA4.0
e sono pubblicate in Open Access su: https://libri.unimi.it/index.php/milanoup.

© 2023 Giulia Lamiani, Michela Maxia, Stefano Iodice, Giovanni Mistraletti
© Milano University Press per la presente edizione

Foto di Stefano Elli, Alessandro Gatti, Samuel Kambu
Gruppo di lavoro: Federica Bonazza, Massimo Walter Rivolta, Elena Vegni

Pubblicato da:
Milano University Press
Via Festa del Perdono 7 – 20122 Milano
Sito web: https://milanoup.unimi.it
e-mail: redazione.milanoup@unimi.it

L'edizione cartacea del volume può essere richiesta
sul sito di SIAARTI - https://www.siaarti.it/
e sul sito di Milano University Press - https://milanoup.unimi.it

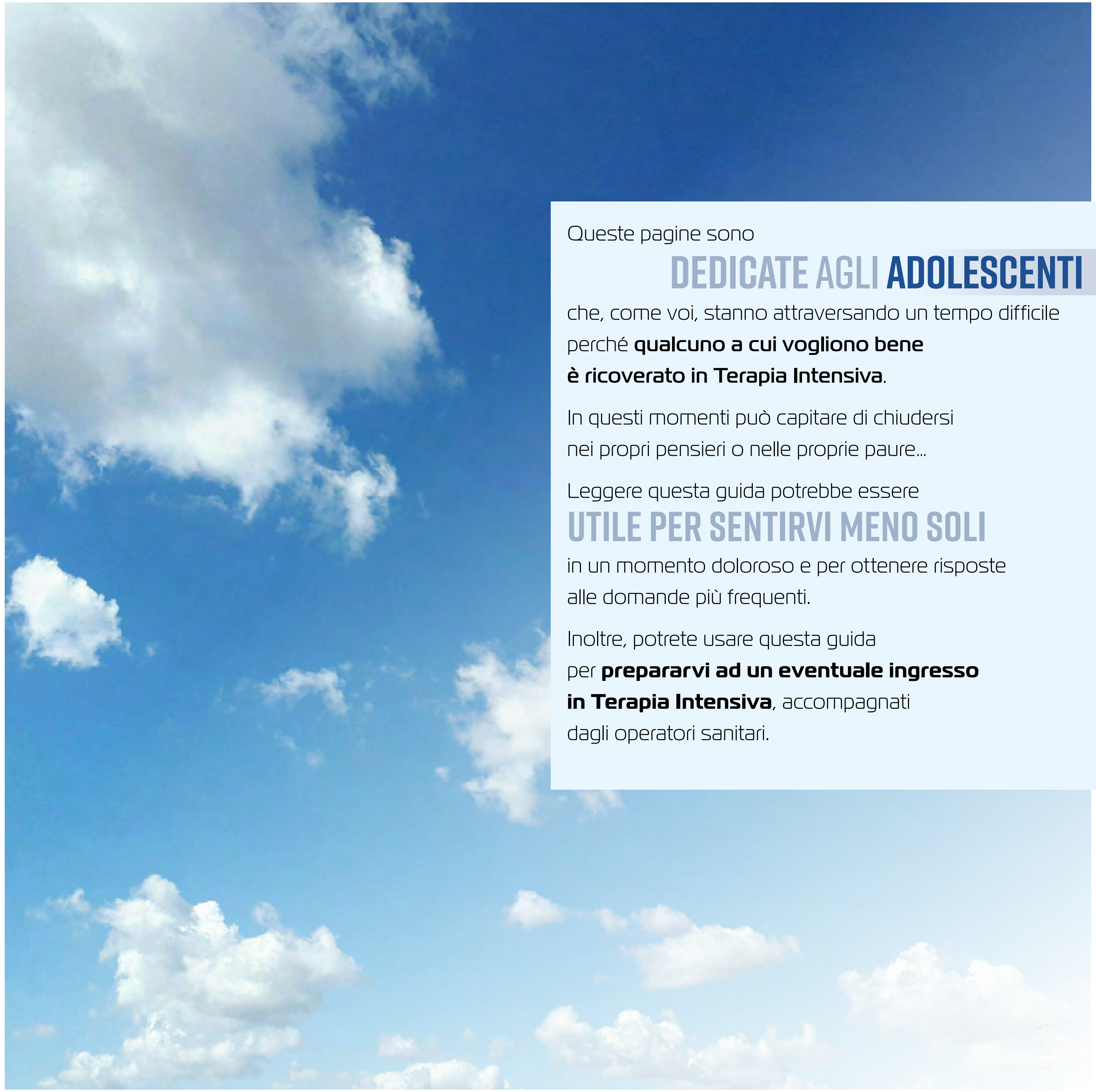

Queste pagine sono
DEDICATE AGLI ADOLESCENTI
che, come voi, stanno attraversando un tempo difficile
perché **qualcuno a cui vogliono bene**
è ricoverato in Terapia Intensiva.

In questi momenti può capitare di chiudersi
nei propri pensieri o nelle proprie paure...

Leggere questa guida potrebbe essere
UTILE PER SENTIRVI MENO SOLI
in un momento doloroso e per ottenere risposte
alle domande più frequenti.

Inoltre, potrete usare questa guida
per **prepararvi ad un eventuale ingresso**
in Terapia Intensiva, accompagnati
dagli operatori sanitari.

COS'È LA TERAPIA INTENSIVA

La Terapia Intensiva (T.I., cioè la Rianimazione) offre cure ad alta specializzazione per aiutare le persone in **pericolo di vita** a superare il momento critico e a recuperare la salute e l'autonomia, quanto più possibile.

Le persone ricoverate in T.I. hanno bisogno che vengano sostenute alcune loro funzioni vitali (circolazione, respirazione, nutrizione...) e che i loro **"parametri vitali"** (battito cardiaco, ossigeno nel sangue, temperatura corporea...) siano controllati costantemente. Per questo vengono utilizzati apparecchi che consentono di capire immediatamente ogni variazione di questi parametri, per intervenire quando è opportuno.

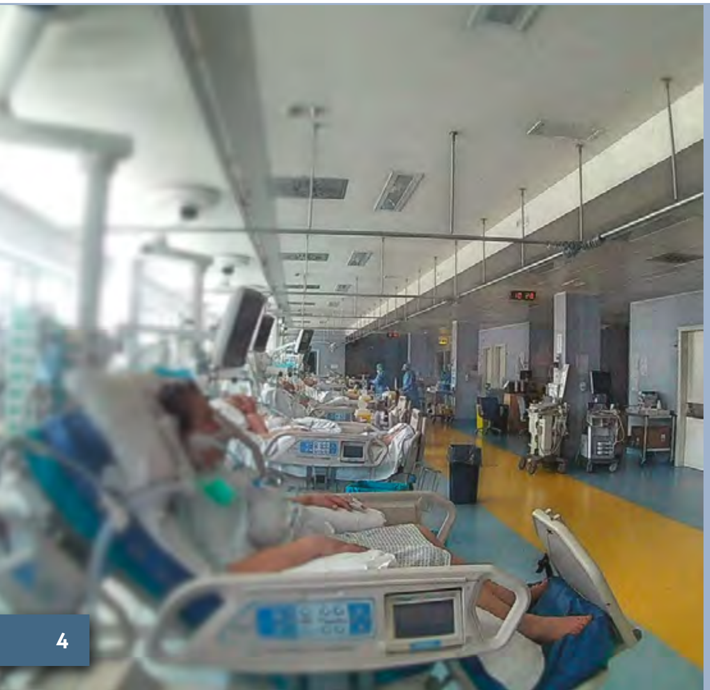

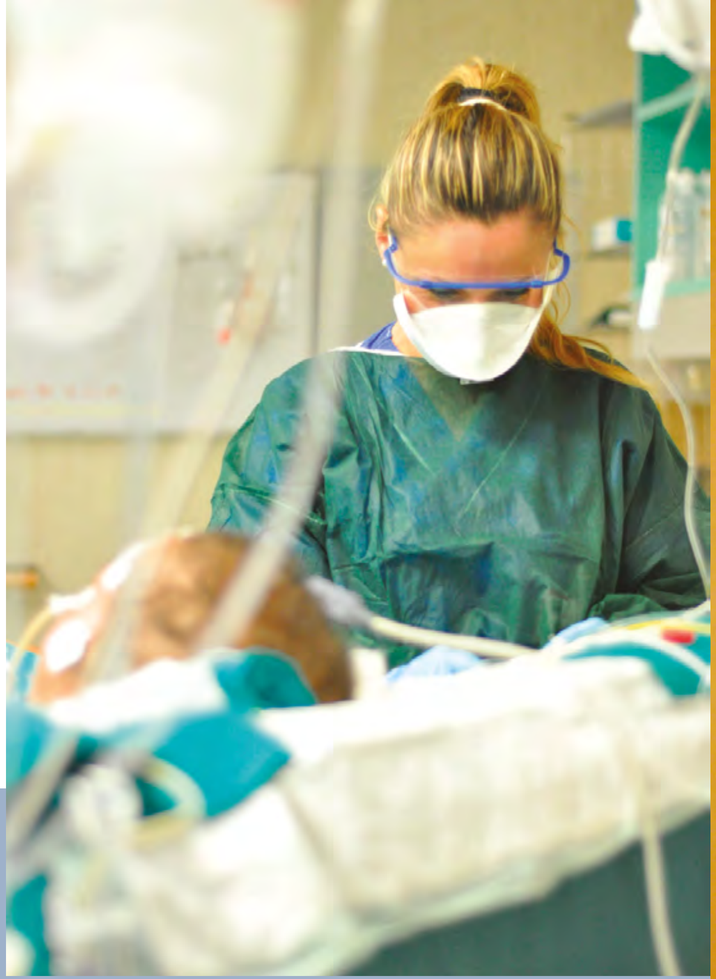

In T.I. il personale sanitario è sempre presente, **24 ore** al giorno per **7 giorni** alla settimana.

Infermieri e medici provvedono a tutte le necessità delle persone ricoverate: fornendo le cure mediche, assicurando il sostegno alle funzioni vitali e curando il rapporto con i familiari del paziente.

I reparti di T.I. sono generalmente costituiti da un unico ambiente protetto ma senza barriere, in modo da consentire una sorveglianza continua e un'assistenza tempestiva, per **garantire le migliori cure**.

REGOLE PER L'INGRESSO

Ogni T.I. ha delle regole sulla presenza dei familiari in visita.

Queste sono le più importanti:

SAPER ASPETTARE

Prima di entrare in T.I. potrebbe esservi richiesto di aspettare in **sala d'attesa**: il personale può essere impegnato in altre urgenze. Appena possibile, un operatore vi accompagnerà in reparto.

RIDURRE LE INFEZIONI

Prima di avvicinarvi al letto del paziente e quando uscirete dalla T.I., **lavatevi sempre le mani** con molta cura, per diminuire il rischio di trasmettere malattie infettive.

RISPETTARE L'INTIMITÀ DEGLI ALTRI

Anche se gli spazi sono aperti, per **rispettare la privacy** delle altre persone ricoverate e delle loro famiglie, evitate di avvicinarvi al loro letto o di leggere le loro cartelle cliniche.

MANTENERE UN AMBIENTE TRANQUILLO

Parlate sempre a voce bassa; ricordatevi di **togliere la suoneria** del cellulare. Un rumore improvviso in un ambiente per malati fragili può essere disturbante.

EVITARE INCIDENTI

Non toccate apparecchi, tubi o qualsiasi altra cosa, neppure se qualcosa vi stupisce o vi preoccupa: potrebbe essere pericoloso per il paziente. Se avete dubbi, **chiedete** a medici o infermieri.

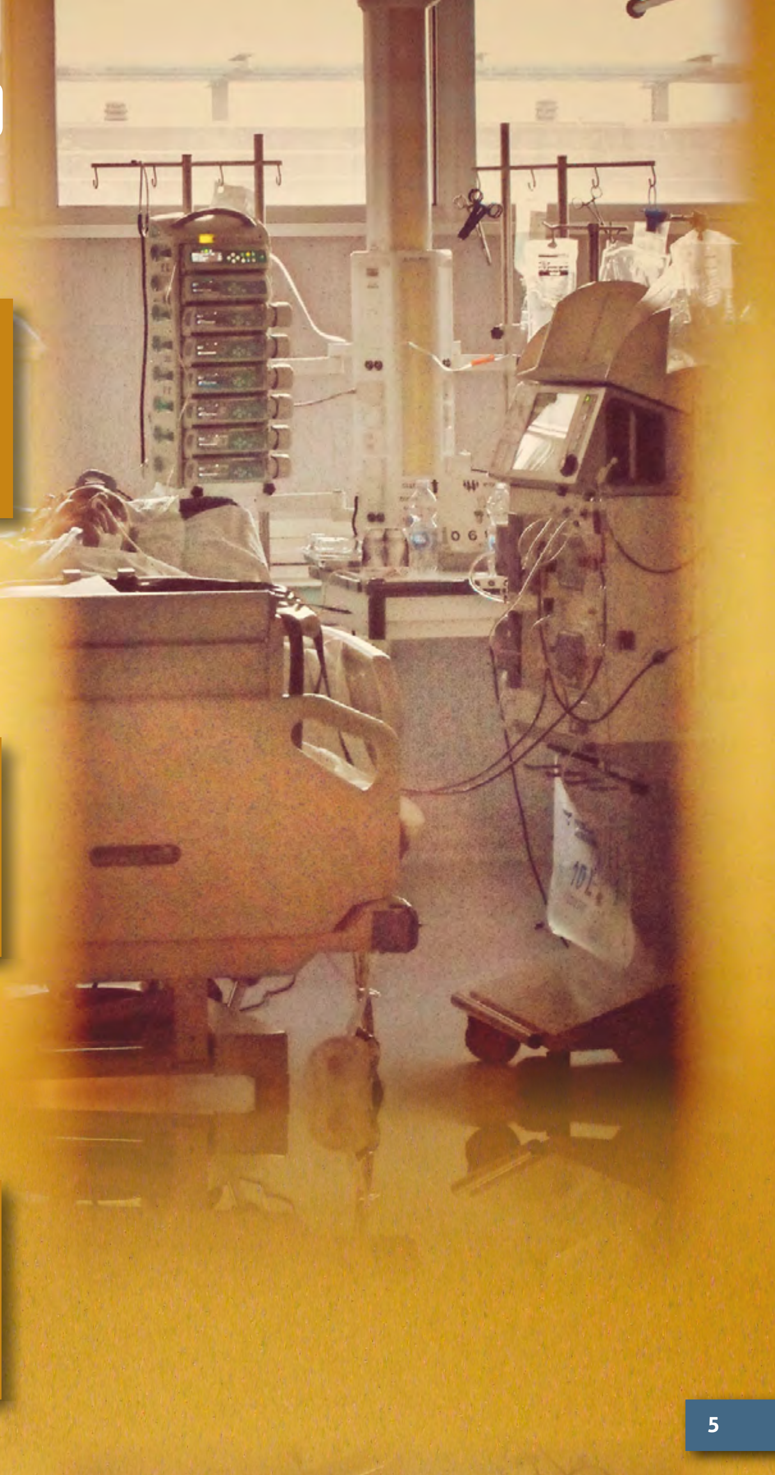

COMPRENDERE L'UNITÀ DI CURA IN TERAPIA INTENSIVA

Questa è una **postazione** di T.I.

Attorno al letto ci possono essere **molte apparecchiature**, essenziali per sostenere le funzioni vitali e aiutare le persone ricoverate a guarire.
Questi strumenti non sono sempre tutti presenti, ma vengono utilizzati solo quando è necessario.

Le apparecchiature possono suonare o illuminarsi all'improvviso; è un modo per **segnalare** agli operatori sanitari che potrebbe essere necessario intervenire o controllarne il funzionamento, ma **non devono farvi spaventare.** Gli operatori sanno distinguere quando c'è un effettivo problema, ed intervengono nel momento appropriato.

Nella stanza di T.I. potreste sentire odore di alcool o disinfettante utilizzati per igienizzare.
La **pulizia** delle superfici e dei materiali è fondamentale per non trasmettere le infezioni ospedaliere!

Se avete dubbi o domande sulla condizione della persona ricoverata, potete **chiedere spiegazioni** al personale sanitario presente. I medici e l'équipe infermieristica saranno a vostra disposizione per rispondere alle vostre domande, spiegarvi cosa accade e a cosa servono le diverse apparecchiature.

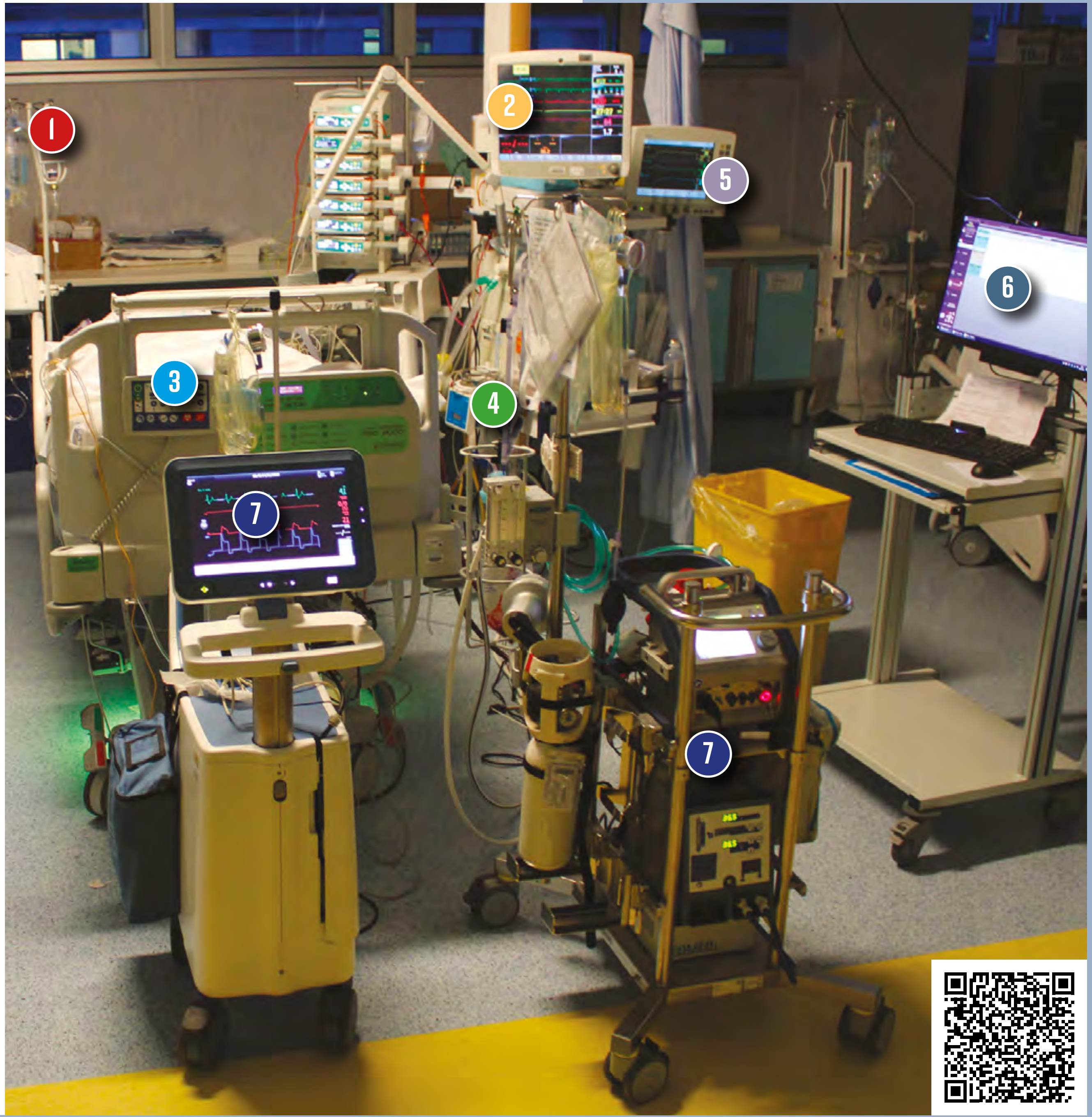

1 **Infusioni endovena (flebo).** È necessario infondere liquidi ai pazienti in maniera costante (farmaci, sostanze nutrienti), per questo si usano sacche sterili collegate a tubicini morbidi (cateteri) che vanno nelle vene.

2 **Monitor.** È uno schermo su cui vengono visualizzati in continuo i parametri vitali (temperatura, pressione arteriosa, frequenza cardiaca...) ed è collegato ad un computer centrale che consente agli operatori di osservare contemporaneamente tutti i pazienti ricoverati. Dal monitor vengono emessi diversi tipi di allarme, ciascuno con un proprio significato, udibili in qualsiasi punto del reparto.

3 **Letto.** Si alza e si abbassa con un telecomando e ha un materasso ad aria. È lontano dalle pareti in modo che sia accessibile, da parte degli operatori sanitari, dai quattro lati. Ha spondine rimovibili per garantire la sicurezza dei pazienti e ruote nel caso siano necessari spostamenti urgenti.

4 **Ossigenoterapia.** Se un paziente ha bisogno di più ossigeno di quello che c'è nell'aria, è possibile dargliene un'aggiunta attraverso degli erogatori posti vicino al letto. L'ossigeno arriva alla bocca e al naso attraverso cannucce nasali, "maschere" facciali o caschi in plastica.

5 **Ventilatore meccanico.** Funziona come una pompa ad aria. Serve per aiutare i pazienti a respirare soffiando ossigeno nei polmoni attraverso tubi che lo collegano alla bocca, al naso, o alla tracheostomia (un accesso praticato nel collo che viene chiuso quando non serve più). In questi casi, non si può temporaneamente parlare, perché l'aria non entra in contatto con le corde vocali.

6 **Cartella clinica.** Su un computer o su una cartella cartacea vengono riportate le informazioni cliniche del paziente: quali terapie sta ricevendo, quali malattie sono ancora presenti e come procede la guarigione.

7 **Contropulsatore aortico e Ossigenazione extracorporea a membrana (ECMO).** Quando il cuore non riesce a pompare abbastanza sangue verso tutto il corpo, si usano delle macchine che aiutano in parte o completamente la sua funzione.

per saperne di più

COME CI SI PUÒ SENTIRE

Le emozioni che si provano in momenti come questi
possono essere molto diverse, contrastanti o confuse.
In questa nuvola ne potreste trovare alcune che state provando anche voi.
Non ci sono emozioni "giuste" o "sbagliate".
Prestare attenzione a quello che si prova
può guidare a comprendere ciò di cui si ha bisogno.

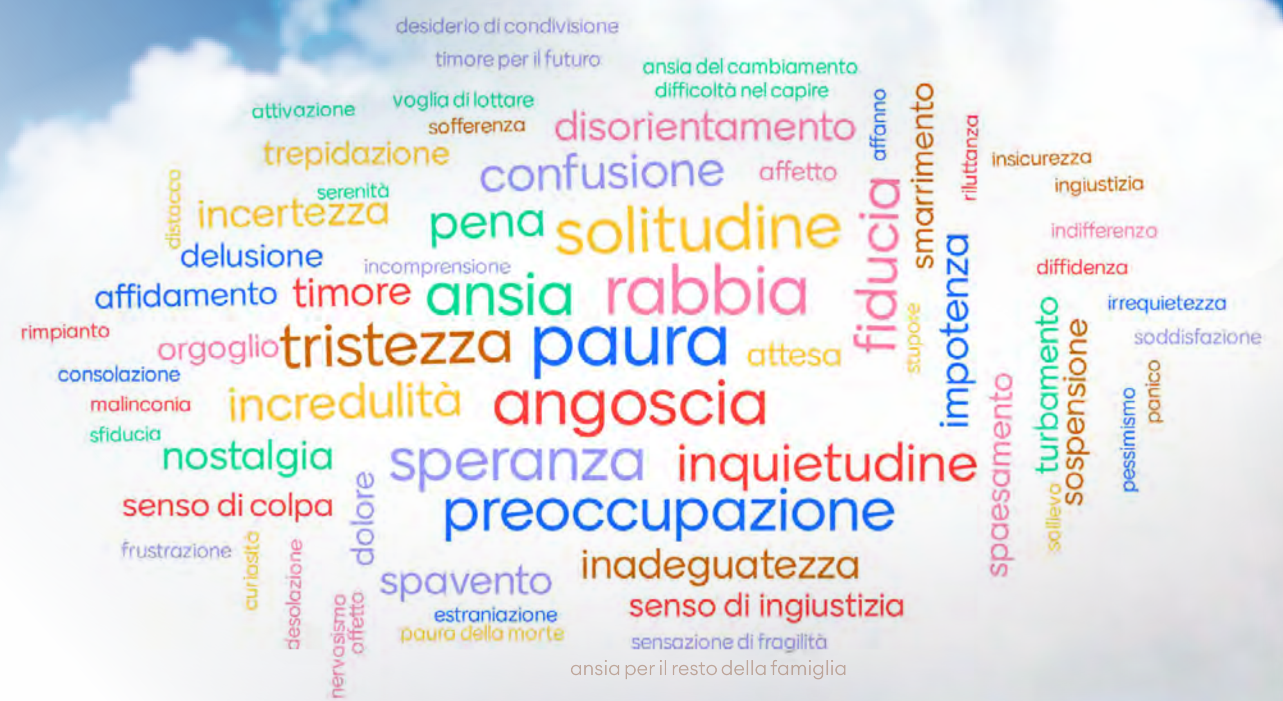

Quando una persona a cui si vuole bene sta soffrendo
o è in pericolo di vita, può capitare di chiudersi
e di tenere dentro le proprie emozioni per paura di far male
o appesantire ulteriormente gli altri familiari.
Lasciare questi sentimenti inespressi, però, può essere faticoso.
Trovare persone fidate (amici, familiari, psicologi)
con cui condividerli può aiutare a sentirsi meno soli.
Anche se **la sofferenza** è unica e soggettiva,
può essere compresa e accolta.

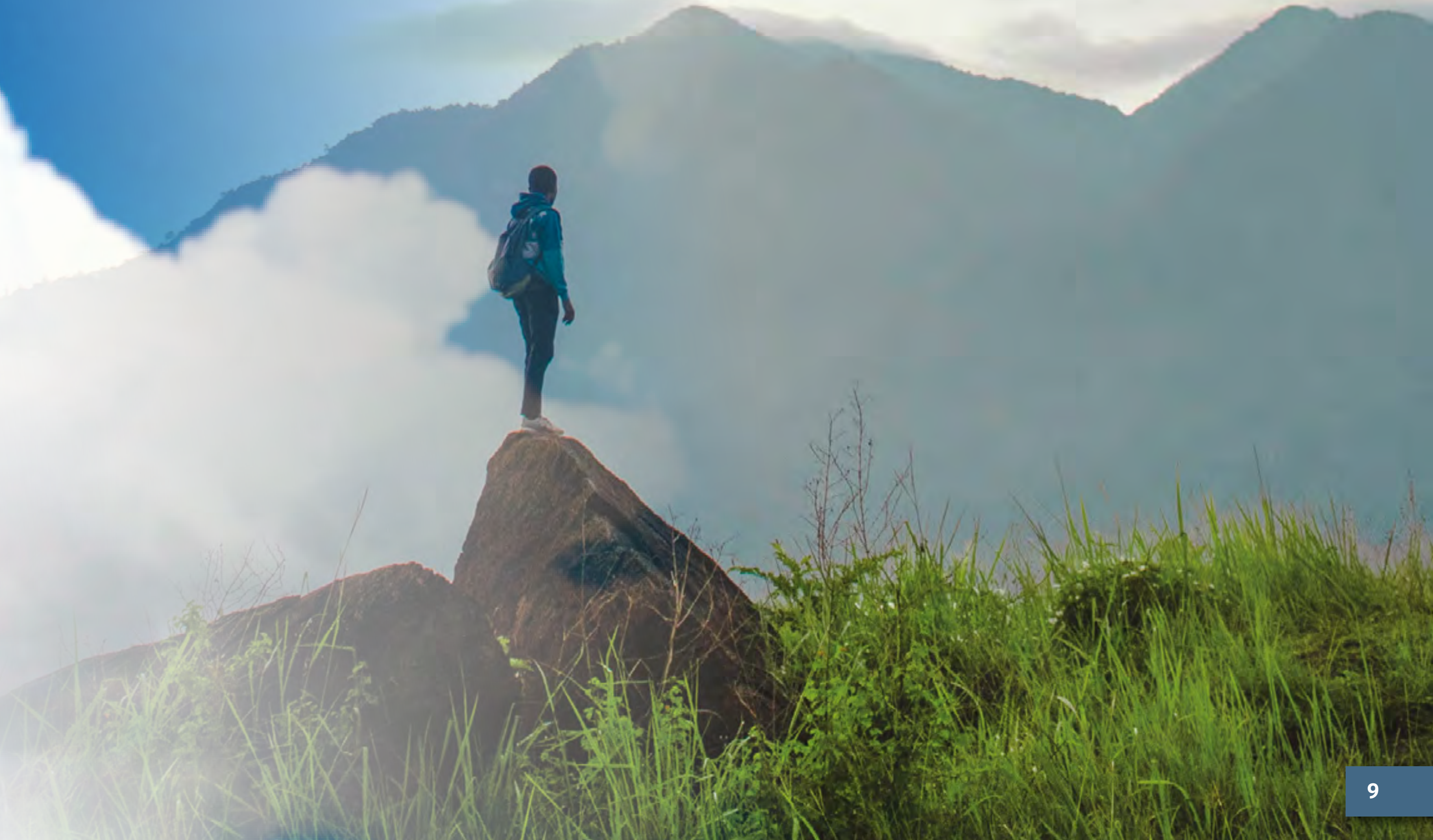

ESPERIENZE DI ADOLESCENTI

"Sinceramente all'inizio il fatto che la nonna fosse in Terapia Intensiva mi ha spaventato perché dal nome non sembrava affatto un posto rassicurante però poi mi sono tranquillizzata perché era controllata da medici esperti e una parte di me ha sempre pensato che mia nonna fosse immortale".

Emanuela, 14 anni

"Il fatto che avessimo il nonno in T.I. ha fatto sì che stessimo tutti più vicini con telefonate e momenti di preghiera. Il fatto di poter comunicare con lui su WhatsApp fino a quando è stato possibile mi ha aiutato."

Enrico, 14 anni

"Il sentimento che prevaleva era la speranza che mio cugino si svegliasse presto. Ci avevano detto che era in coma farmacologico e ricordo che non sapevo neanche cosa volessero dire quelle parole. Era estate e abbiamo ospitato da noi l'altro mio cugino. Io e le mie sorelle dovevamo essere forti per cercare di farlo distrarre. Ricordo che era un continuo essere in attività per non pensare a quello che stava succedendo. A chi dovesse passare in questa situazione mi sentirei di consigliare di esternare i propri sentimenti perché è normale stare male e piangere. Se non si riesce con i propri cari, cercate un amico con cui poterlo fare."

Michela, 17 anni

"In quei giorni ho percepito una preoccupazione costante, un vuoto incolmabile, ma anche un senso di minima speranza. Suggerirei di provare ad avere fiducia nel personale medico, di porre qualsiasi domanda senza vergogna e di accettare le notizie negative, perché non sempre è possibile combattere il destino."

Valerio, 16 anni

"Stavo malissimo. Vaffanculo all'infarto di mio padre. Quando me lo han detto non volevo crederci... mi sono chiesto perché fosse successo proprio a mio padre... Quando si è ripreso si vedeva che era spaesato ma che combatteva come un leone. In quel periodo mi ha aiutato la vicinanza di mia mamma e anche degli amici. Andare a trovare mio padre in motorino in ospedale era il momento più atteso della giornata, e non ci andavo mai da solo perché c'era sempre un compagno di classe o di basket, che mi accompagnavano a turno... sono stati dei bomber"!

Giorgio, 15 anni

"Il consiglio che do è quello di trovare il giusto equilibrio tra speranza e rassegnazione. Non bisogna pensare che è impossibile che questa persona muoia, ma neanche abbandonare l'idea che possa guarire. Inoltre, consiglio di passare più tempo possibile con la persona cara e cercare per quanto si può di vivere all'esterno del reparto una vita normale per distrarsi."

Nicole, 14 anni

"Ero quasi incredula, non realizzavo bene la situazione. Consiglierei di affidarsi e circondarsi dell'amore della famiglia, perché è una cosa che si affronta insieme."

Giulia, 15 anni

"All'inizio non volevo entrare in Terapia Intensiva e poi non sapevo neanche se si poteva. Dopo 4 giorni sono entrato ma mio padre non poteva ancora parlare... Sarebbe meglio non trovarsi in questa situazione. Ma se poi ci finisci dentro, tiri fuori delle risorse che pensavi di non avere. A me è successo di riscoprire l'importanza degli amici, che ti fanno sentire come una famiglia"

Luca, 15 anni

Se vorrai, potrai condividere la tua storia

...oppure leggere quella di altre persone

COSA POTETE FARE VOI

Davanti a cattive notizie e a sofferenze improvvise **non si può essere preparati**. Sentirsi impotenti e fragili è normale, ma ciò non esclude la possibilità di fare qualcosa per la persona ricoverata, per voi stessi e per chi vi è vicino.

...PER LA PERSONA RICOVERATA

Far sentire la vostra presenza è prezioso. Per la persona ricoverata vedere volti o sentire voci familiari può essere un importante **raccordo con la vita fuori dall'ospedale**. Potete raccontare ciò che accade fuori o portare oggetti personali (libri, giornali, riviste...) che le permettano di sentirsi al sicuro in un ambiente nuovo e sconosciuto; saranno gli infermieri ad utilizzarli nei momenti giusti.

Potreste portare **foto o disegni** da appendere, la **musica preferita** o uno smartphone/tablet per comunicare o vedere film. Medici e infermieri sono disponibili a conoscere i vostri cari anche attraverso le vostre parole. Questo è un aiuto importante per facilitare la **personalizzazione delle cure**.

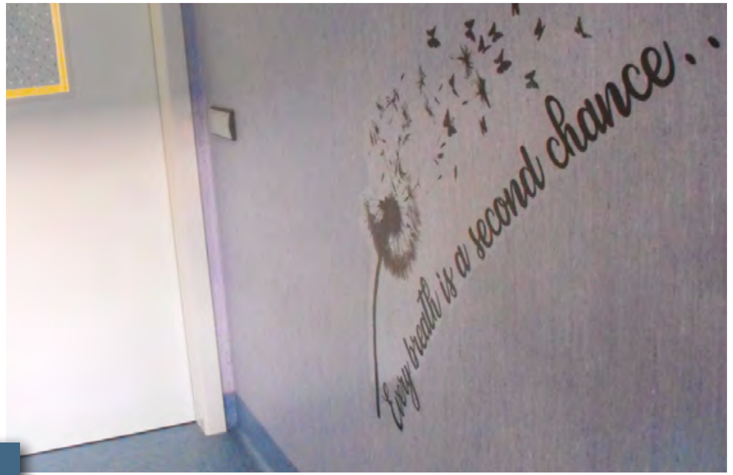

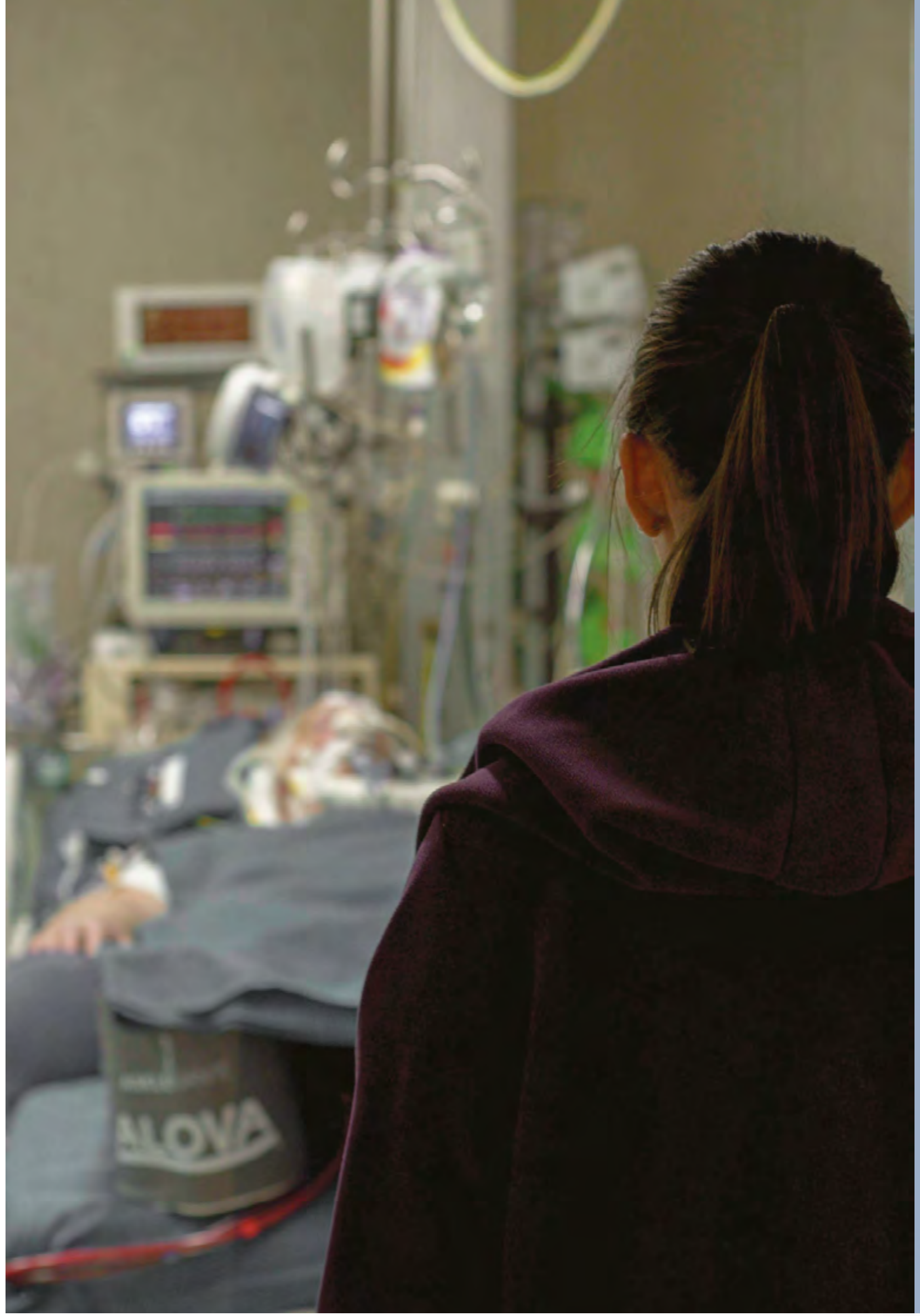

...PER VOI STESSI

In questa situazione difficile è importante fare **rifornimento di energie buone** che vengono dalla vita fuori dall'ospedale.

La famiglia e gli amici sono risorse preziose: potete **parlare di quello che state provando**, di ciò che vi preoccupa o che vi farebbe stare meglio. Non spaventatevi delle vostre emozioni, datevi tempo.

Ci si può concedere delle **attività per svagare la mente:** lo sport, un film, una pizza, una passeggiata nella natura o una serata a ballare. Potete scrivere una canzone o fare un dipinto: **impegnarsi in attività espressive** può far stare meglio.

Dosate le vostre energie: è importante tenere insieme i diversi aspetti della vita, che sono stati travolti da questa situazione.

Se sentite di essere in difficoltà, se le emozioni vi travolgono, se provate rabbia o tristezza o voglia di piangere in un modo che è nuovo e strano per voi, ricordate che i medici e gli infermieri sono a vostra disposizione per mettervi in contatto con chi vi saprà ascoltare. **Chiedere sostegno,** in questi casi, è un atto di coraggio.

...PER CHI VI È VICINO

Quando **emozioni così intense** - come quelle che potreste provare in questo momento - non vengono espresse, aleggiano comunque in famiglia, a scuola e con gli amici. **Condividere quello che si prova** può essere utile sia a voi, sia a chi vi vuole bene.

In famiglia è possibile trovare modi per sentirsi vicini attraverso **azioni semplici e concrete**, come guardare un film seduti sullo stesso divano o preparare la cena insieme.

A volte può essere necessario **riorganizzare la routine familiare per aiutarsi a vicenda:** in questi casi, potreste cucinare, riordinare la stanza o farvi carico di piccole faccende quotidiane.

Si può anche provare ad **essere disponibili** per chi vi sembra maggiormente in difficoltà, come ad esempio i vostri fratelli minori o i nonni. Ci sono molti contesti in cui potete essere una risorsa preziosa! Non abbiate timore di confrontarvi per comprendere come meglio agire.

Gli sguardi, gli abbracci, il semplice **"stare vicino"** possono essere più importanti delle parole...

MUSICA ED EMOZIONI

PLAY

La musica può far **risuonare le emozioni** e **consolare** anche nei momenti più bui, oppure può essere una valvola di **sfogo**. Ognuno ha la propria musica preferita, ed è questa che vi consigliamo di ascoltare; ma poiché la malattia, la mancanza di una persona cara e la paura della morte sono esperienze comuni che tanti artisti hanno messo in musica, vi suggeriamo alcune canzoni che crediamo possano **farvi sentire meno soli**.

MODÀ - FEAT. JARABEDEPALO
COME UN PITTORE

ARTISTI UNITI
DOMANI 21.04.2009

JOVANOTTI
FANGO

COLDPLAY
DADDY

BLANCO
BLU CELESTE

GIORGIA
GOCCE DI MEMORIA

FRANCESCO GABBANI
ETERNAMENTE ORA

SPECIALIZZANDI RIANIMAZIONE SAN PAOLO
MOSAICI BIANCHI

Se volete **mandarci "le vostre canzoni"** potete andare qui:

Con le canzoni suggerite da altri ragazzi e ragazze che – come voi – hanno avuto una persona cara ricoverata in T.I., abbiamo creato questa **playlist:**

ESPERIENZE DI CHI STA DALL'ALTRA PARTE

PERCHÉ HO SCELTO QUESTO LAVORO

Quando avevo 22 anni, mentre ero studentessa al quarto anno di medicina, fui toccata da una storia incredibile.
Il **ragazzo di cui ero innamorata** era in piscina a lavorare. Era luglio, lui faceva il bagnino, ed ebbe improvvisamente un **arresto cardiaco**.
Ancora oggi mi ricordo le telefonate concitate e il racconto di frammenti di quanto succedeva: il massaggio cardiaco e la defibrillazione, l'arrivo dei soccorsi, poi l'automedica che proseguiva le cure in una **folle corsa verso l'ospedale**.

Tutti fummo travolti da un dolore mai provato prima. Io sentivo la responsabilità e la frustrazione di non essere stata lì... anche se non ero ancora un vero medico, ma solo una studentessa del quart'anno.
Per la prima volta **la vita mi sembrava attaccata a un filo** e scoprivo giorno dopo giorno una parte di me sconosciuta, buia, triste e impaurita, che potevo solo accettare e cercare di indirizzare perché mi facesse meno male, ma che non potevo annullare. Cercai di andare a trovarlo in Rianimazione, ma non si poteva entrare.

Morì dopo una settimana di Terapia Intensiva a causa dell'evoluzione del coma post-anossico; i suoi genitori decisero di **donare i suoi organi**.
Per molti mesi non riuscii più ad andare in piscina senza pensare a quello che gli era successo. Poi decisi di entrare nella Pubblica Assistenza; facevo già del volontariato, ma questo era diverso. Mi serviva per cercare di **comprendere** cosa fosse effettivamente successo, e per **accettare** l'inevitabile solo dopo aver fatto tutto il possibile.

Mi interrogai a lungo per cercare di capire cosa potessi trarre da questa esperienza.

Il dolore mi rendeva incredula.
Davvero non si poteva fare niente di meglio per lui?
La ricerca medica, le terapie più all'avanguardia... si erano rivelate tutte inefficaci. Nell'anno seguente dovevo decidere in quale disciplina medica chiedere di fare la mia tesi di laurea. Ero incerta fra neuropsichiatria infantile, radiologia interventistica, endocrinologia, oppure anestesia e rianimazione.

Quel periodo fu tanto triste, quanto prezioso per **fare luce sulle priorità della mia vita**.
Certo, tutti i medici possono essere utili, ma una rianimatrice può fare la differenza fra la vita e la morte, può combattere l'ultima battaglia in modo saggio e competente, prima di comprendere che non c'è più nulla da fare.
Il mio è un mestiere duro, meraviglioso e difficilissimo.
Io credo che sia il più bello del mondo, ma sono anche consapevole che espone a grandi rischi: di indurirsi emotivamente, di diventare cinici o di perdere la speranza.

Essendo sempre a contatto con storie drammatiche, ho imparato a **pensare prima di giudicare un comportamento altrui**, ho cercato di vivere insieme agli altri con maggiore empatia, ho visto molte persone che modificavano in meglio il loro stile di vita dopo aver rischiato di morire.

Il rianimatore può essere un faro nella tempesta.
Un faro fragile, umano, ma per questo anche credibile.
E porta la consapevolezza – come la stella del mattino – che non importa quanto sia stata buia e lunga la notte: a un certo punto, **l'alba arriverà.**

Martina, dottoressa rianimatrice

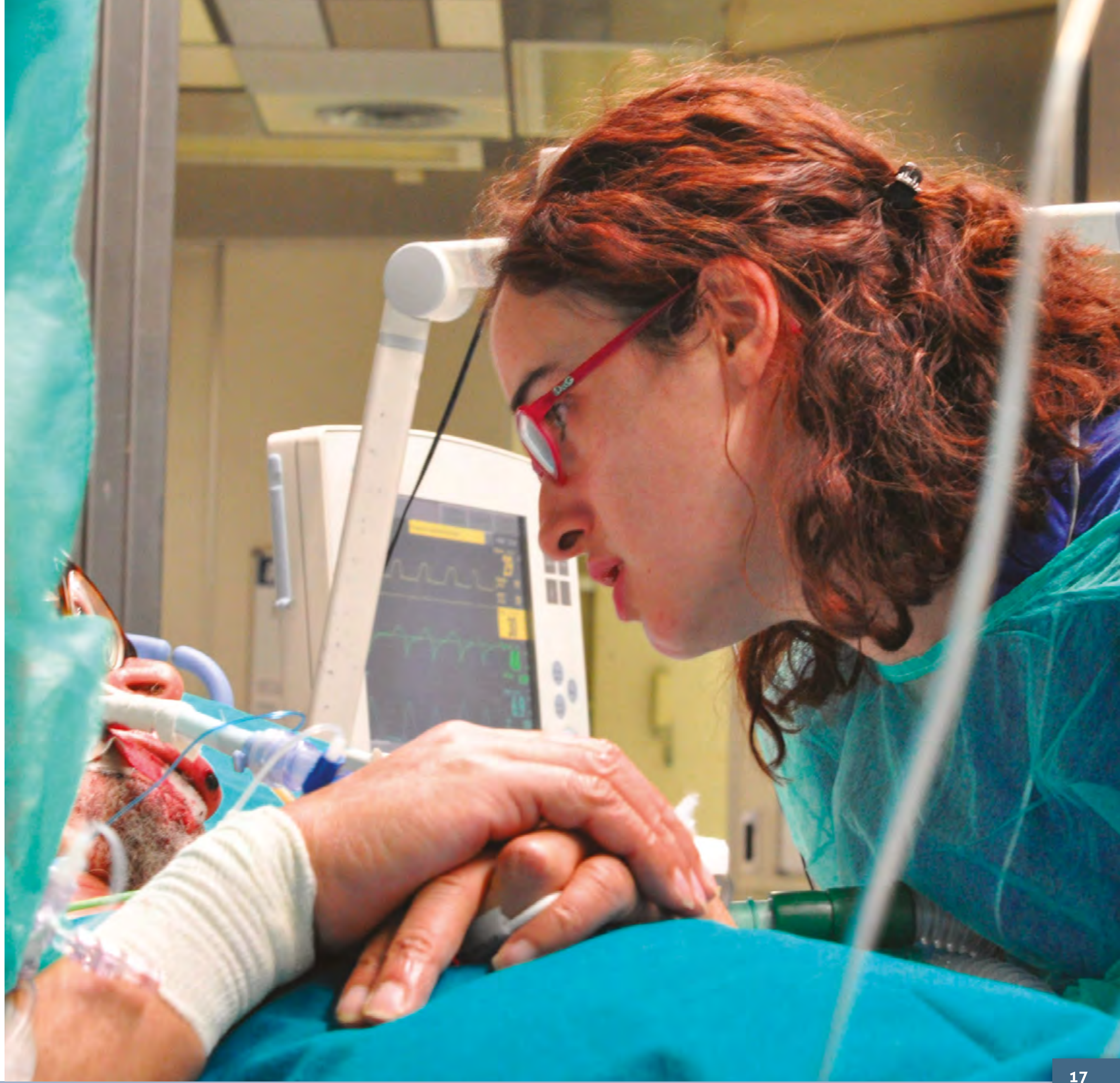

ESPERIENZE DI CHI STA DALL'ALTRA PARTE

ONDE

È notte.
Le **luci** del reparto sono spente.
In sottofondo i rumori sommessi delle macchine in funzione, dei flussi d'aria dei respiratori, di allarmi isolati che richiedono minimi accorgimenti.

I **monitor** delimitano ogni postazione.
Queste sono segnate da un numero.
Un posto letto.
Una persona.

C'è il **signor Luca**. Ha 46 anni.
Percorreva in moto la strada per tornare a casa, poi un'automobile è spuntata all'improvviso, e da lì, il buio. Ha avuto un forte trauma cranico e diverse fratture a braccia, gambe, bacino.

È sedato profondamente e i farmaci in infusione controllano il dolore. Un'ampia fasciatura gli copre la testa, il ventilatore spinge aria nei polmoni aiutandolo a respirare, un dispositivo sostituisce temporaneamente il funzionamento dei suoi reni.
Le onde sui monitor salgono e scendono dando forma ai parametri vitali.
Qui **la vita si mostra in tutta la sua fragilità**.
I cambiamenti clinici non di rado richiedono giorni, settimane, mesi... In molti casi è il tempo stesso la cura.
Ieri ho visto **suo figlio**. Aspettava con la mamma, seduto in sala d'attesa.

-"*È possibile entrare?*", mi chiede sottovoce.
-"Certo! Come ti chiami?"
-"*Michele.*"
-"Vieni Michele, ti accompagno..."

Attraversiamo il corridoio.
Scorge gli altri letti prima di arrivare a quello di suo papà.
Ci avviciniamo.

Lo guarda in viso senza dire una parola, poi sospira profondamente.
-"È ancora sedato. È normale che sia così..."
Annuisce. Incrocia le braccia e quasi si allontana dal letto.

-"Come ti senti?", gli chiedo.
Alza le spalle, poi scuote la testa.
-"*Io... non lo so.* ***Non sembra nemmeno lui***.", risponde dopo qualche istante.

- "Puoi avvicinarti... ", gli dico.
Rimane fermo, indeciso su cosa fare.
-"*Ci sono tanti tubicini e fasciature.*
Non vorrei fare casini."
-"Non preoccuparti. Puoi toccargli la mano in sicurezza...", lo incoraggio.

Accosta la mano a quella di suo papà.
La sfiora, la accarezza e infine gliela stringe.

La paura di avere un tuo caro in un letto d'ospedale, spaventato, disorientato, circondato da persone che non conosce. La paura che provi dolore.

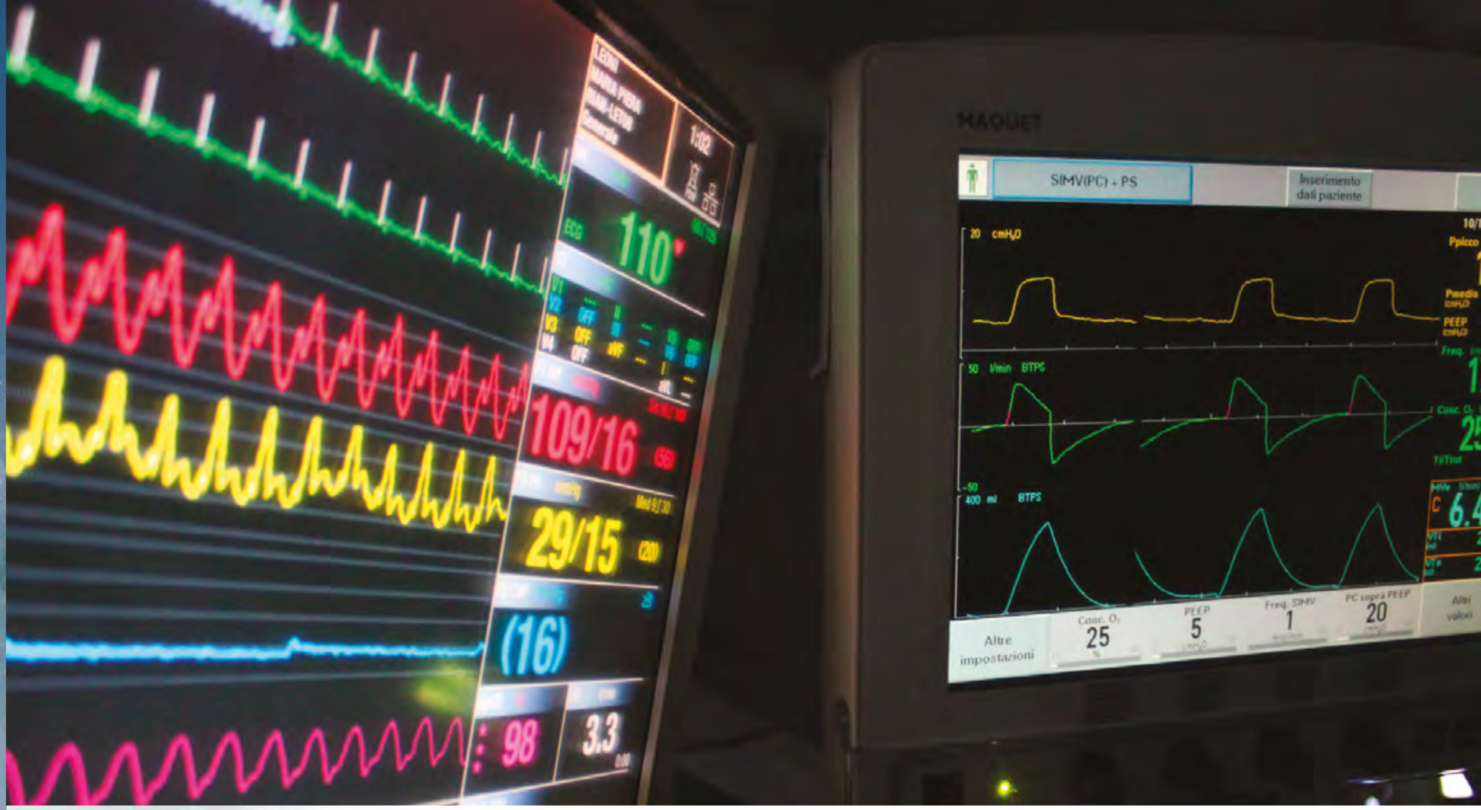

L'attesa di una chiamata e il timore che i medici non abbiano cose buone da dire.
L'incredulità che tutto questo sia accaduto davvero. Come è potuto succedere?
A volte un **piccolo dettaglio** è capace di sconvolgerti la vita. Perché proprio a lui?
Di certo non lo meritava...
Chi può mai meritarselo?

In Terapia Intensiva, come infermiere, mi trovo spesso **di fronte alle mie più grandi paure**.
Ho imparato ad accoglierle, a conviverci, a esprimerle, e scopro che sono **le paure di tutti**.
Vivo con la consapevolezza che tutto questo possa accadere a chiunque da un momento all'altro.
Anche a chi amo.
Anche a me stesso.

E allora mi fermo e **comincio a valorizzarli, i piccoli dettagli**. Perché è vero che possono sconvolgerti la vita ma anche fartela apprezzare.
Sono questi i momenti in cui **mi sento davvero vivo**.
Dò ancora un'occhiata alla postazione, ai macchinari, al signor Luca.
Le onde sui monitor danno forma ai parametri vitali.

Per ogni discesa c'è sempre una risalita.
In molti casi, è solo una questione di tempo.

Stefano, infermiere di Terapia Intensiva

Abbiamo realizzato questa guida pensando agli **adolescenti dai 14 ai 17 anni** che hanno una persona cara ricoverata in Terapia Intensiva.

Tutti noi (medici, infermieri, psicologi...) desideriamo **esservi d'aiuto** perché non vi sentiate soli in questo momento di legittima preoccupazione.

Il nostro impegno si unisce a quello di tanti reparti di Terapia Intensiva, in Italia e nel mondo, per "**umanizzare**" le cure offerte ai malati e ai loro familiari.

Ringraziamo tutti gli operatori sanitari e gli adolescenti che hanno condiviso le storie qui raccontate.

Questa pubblicazione fa parte di un più ampio progetto di ricerca-azione (Progetto MinViTI) avente l'obiettivo di migliorare il benessere psicologico dei minori che hanno un familiare ricoverato in Terapia Intensiva.
Il progetto è stato finanziato attraverso un Seal of Excellence (SoE) dal Piano di Sostegno alla Ricerca (2020) dell'Università degli Studi di Milano.

La guida è stata realizzata in collaborazione con il **Progetto Intensiva.it**, un'iniziativa scientifica promossa e supportata da SIAARTI, Aniarti, AAROI-EMAC.

Per ulteriori domande contattateci: **info@intensiva.it** oppure consultate il sito **www.intensiva.it**

INTENSIVA.it
Una strada da condividere

SIAARTI
PRO VITA CONTRA DOLOREM SEMPER

AAROI EMAC
Associazione Anestesisti Rianimatori Ospedalieri Italiani Emergenza Area Critica

aniarti
associazione nazionale infermieri di area critica

HEROIC bundle
HUMANIZATION TO ENHANCE RECOVERY ON INTENSIVE CARE
